# *i-MoMCARE*: Innovative Mobile Technology for Maternal and Child Health Care in Cambodia—study protocol of a cluster randomized controlled trial

**DOI:** 10.1186/s13063-023-07724-z

**Published:** 2023-10-26

**Authors:** Chan Hang Saing, Mengieng Ung, Sovanthida Suy, Sreymom Oy, Chhavarath Dary, Esabelle Lo Yan Yam, Sophea Chhorn, Michiko Nagashima-Hayashi, Dyna Khuon, Sovatha Mam, Rattana Kim, Vonthanak Saphonn, Siyan Yi

**Affiliations:** 1 Saw Swee Hock School of Public Health, National University of Singapore and National University Health System, 12 Science Drive 2, #10-01, Singapore, 117549 Singapore; 2 University of Health Sciences, Phnom Penh, Cambodia; 3 College of Health and Medicine, Australian National University, Canberra, Australia; 4 National Maternal and Child Health Center, Phnom Penh, Cambodia; 5 KHANA Center for Population Health Research, Phnom Penh, Cambodia; 6 Public Health Program, College of Education and Health Sciences, Touro University California, Vallejo, CA USA

**Keywords:** Community health workers, Digital health intervention, Maternal and child health, Implementation research, Low- and middle-income country, Asia

## Abstract

**Background:**

The Government of Cambodia established the village health support groups (VHSGs) in 2003 to facilitate primary healthcare activities, including maternal and child health (MCH) services. However, VHSGs face several challenges that hinder them from performing optimally, including a lack of regular structured training and remuneration and limited and inconsistent support and supervision from the health centers (HCs). This implementation research aims to develop, implement, and evaluate a digital health intervention to improve the performance of VHSGs through better support and supervision and increase the MCH service coverage in rural Cambodia.

**Methods:**

i-MoMCARE, a two-arm cluster randomized controlled trial, will be conducted between 2022 and 2025. Five operational districts (ODs) have been randomized to an intervention arm and the other five ODs to the control arm. The intervention will last for 24 months. Around 200 VHSGs in the intervention arm will be equipped with a mobile application as a job aid and 20 HC staff with a web interface to improve support and supervision of VHSGs. The potential beneficiaries will include pregnant women, mothers, and children under 2 years old. We will measure the outcomes at baseline and endline. The primary outcomes will consist of a composite MCH index constructed from maternal and newborn care indicators, child immunization, and treatment of under-two children. Secondary outcomes will include coverage of selected MCH services. We will conduct the intention-to-treat and per-protocol analyses. We will conduct qualitative interviews with selected beneficiaries and stakeholders to evaluate the intervention’s acceptability, feasibility, and scalability. We will also conduct a cost-effective analysis using decision-analytic modeling incorporating a societal perspective that explores different time horizons, intervention effects, and when scaled up to the national level.

**Discussion:**

i-MoMCARE is expected to increase MCH service access and coverage in rural Cambodia. It will contribute to advancing digital health use in primary healthcare interventions, which remains in its infancy in the country. Furthermore, the study findings will be a valuable addition to a growing body of literature on the effectiveness and feasibility of mobile health to improve coverage of MCH services in rural low- and middle-income country settings.

**Trial registration:**

ClinicalTrial.gov NCT05639595. Registered on 06 December 2022.

**Supplementary Information:**

The online version contains supplementary material available at 10.1186/s13063-023-07724-z.

## Background

Cambodia’s maternal mortality ratio (MMR) dropped from 488 in 2000 to 168 in 2016 and 154 in 2021 per 100,000 live births [1]. However, the figure remains notoriously higher than that in other countries in Southeast Asia [2]. Further effort is required to achieve the global MMR target in the Sustainable Development Goals at less than 70 per 100,000 live births by 2030 [3]. Likewise, the infant mortality rate (IMR) substantially decreased by 67%, from 79 deaths in 2000 to 26 deaths in 2016 and 12 in 2021 per 1000 live births [1]. The rate, however, remains higher than that in the neighboring countries, except for Laos (40 deaths per 1000 live births) and Myanmar (39 deaths per 1000 live births) [2].

In Cambodia, despite the drop in the country’s average MMR and IMR, urban and rural disparities persist within the country, stemming from inequity in health service accessibility and utilization, making maternal and child death a remaining public health priority [4, 5]. The primary underlying factors associated with maternal and child deaths include inadequate affordable and accessible primary healthcare services, poor-quality services and hygiene, and a lack of skilled health staff [6]. No woman or child should suffer or die from preventable causes. However, many children’s and mothers’ lives are being lost, especially at birth and during the first month of life [7], especially in rural areas of the country. Increasing coverage of and access to maternal and child health (MCH) services in rural areas is imperative to overcome the gaps in MCH care and improve universal health coverage (UHC).

Over the years, various initiatives and interventions have been put in place by Cambodia’s Ministry of Health (MOH), aiming to reduce maternal and child mortality across the country. The interventions are in line with the 2010–2015 Fast-Track Initiative Road Map for Reducing Maternal and Newborn Mortality [8], the 2017–2020 National Strategy for Reproductive and Sexual Health in Cambodia [9], and the 2016–2020 Emergency Obstetric & Newborn Care Improvement Plan [10]. These guiding papers outline effective initiatives to save the lives of women and children in Cambodia. Despite successful interventions, limited human and financial resources are a significant constraint in expanding these life-saving health initiatives that may effectively reduce maternal and newborn mortality, especially in hard-to-reach and resource-limited settings.

In recent years, tapping on the breakthrough of technological advancement, digital health—the broad scope of which includes mobile health (mHealth), health information technology, wearable devices, telehealth and telemedicine, and personalized medicine—has become the mainstream in the public health arena [11]. Digital health has been used to improve MCH in the Global North, such as in Canada [12] and Australia [13], and in the Global South, including China [14], India [15], and Sub-Saharan Africa [16, 17]. Digital health interventions have successfully reduced maternal and child deaths, especially in low- and middle-income countries (LMICs), by providing job aids to primary healthcare workers.

The Innovative Mobile Technology for Maternal and Child Health Care (*i-MoMCARE*) is a digital health intervention aiming to increase the coverage of and access to MCH services for pregnant women and their children in rural communities in Cambodia. We will fully adapt the Innovative Mobile-phone Technology for Community Health Operations (ImTeCHO), an innovative model developed, successfully implemented, and evaluated by SEWA Rural in Gujarat, India [18]. This study will build on Cambodia’s well-established and well-structured healthcare system, increased Internet coverage, and the sharp rise in smartphone adoption [19] to implement innovative mobile technology intervention. On the one hand, *i-MoMCARE* will provide village health support group (VHSG) access and training on mobile-based job aid (the mobile application) to facilitate their work with pregnant women and mothers in the villages. Specifically, with the mobile application, VHSGs can register pregnant women and mothers, schedule health care appointments, develop a digital record of the medical history of pregnant women and mothers, show educational videos to pregnant women and mothers, and alert HC staff on high-risk cases. On the other hand, *i-MoMCARE* will offer health center (HC) staff access to the web interface to track VHSGs’ activities and clients’ medical history and screen for complications and referrals.

VHSGs are the bedrock of Cambodia’s health system, particularly where access to health services is limited and trained health professionals are scarce. They are central in providing and facilitating healthcare services to people, including pregnant women and under-five children living in rural communities. In Cambodia, long distances to health facilities and insufficient education for women of reproductive age are among the critical determinants of poor health outcomes, predominantly in rural communities where the demand for MCH services is higher than in urban areas [20, 21]. VHSGs could help overcome the long distance to health facilities with mobile technology. Hence, *i-MoMCARE* will improve women’s understanding and access to antenatal care (ANC), safe delivery by skilled health personnel, postnatal care (PNC), and child immunization, reducing high-risk cases and maternal and child deaths. This study is the first to be implemented in Cambodia, contributing to advancing digital health use in MCH interventions, which remain in their infancy.

The general objective of this study is to develop, test, implement, and evaluate the applicability and efficacy of the *i-MoMCARE* to support VHSGs and HC staff in rural communities to increase the coverage of and access to MCH services in Cambodia. The specific objectives of this study include the following: (1) to identify gaps in MCH care through conducting a literature review, consulting with local stakeholders, and qualitative need assessments and gap analyses to understand the barriers to accessing MCH services provided or facilitated by VHSGs; (2) to identify technology components that provide resolutions to fill in the gaps in MCH care services; (3) to pilot and qualitatively evaluate the operational delivery of the *i-MoMCARE* intervention; (4) to implement the *i-MoMCARE* intervention among VHSGs and HC staff; (5) to qualitatively evaluate the acceptability, feasibility, and usefulness of the *i-MoMCARE* intervention; (6) to quantitatively evaluate the effectiveness of *i-MoMCARE* in improving the MCH coverage, MMR, IMR, and other key indicators; and (7) to evaluate the cost-effectiveness of *i-MoMCARE* for adopting and scaling up the intervention in Cambodia.

## Methods

**Fig. 1:**
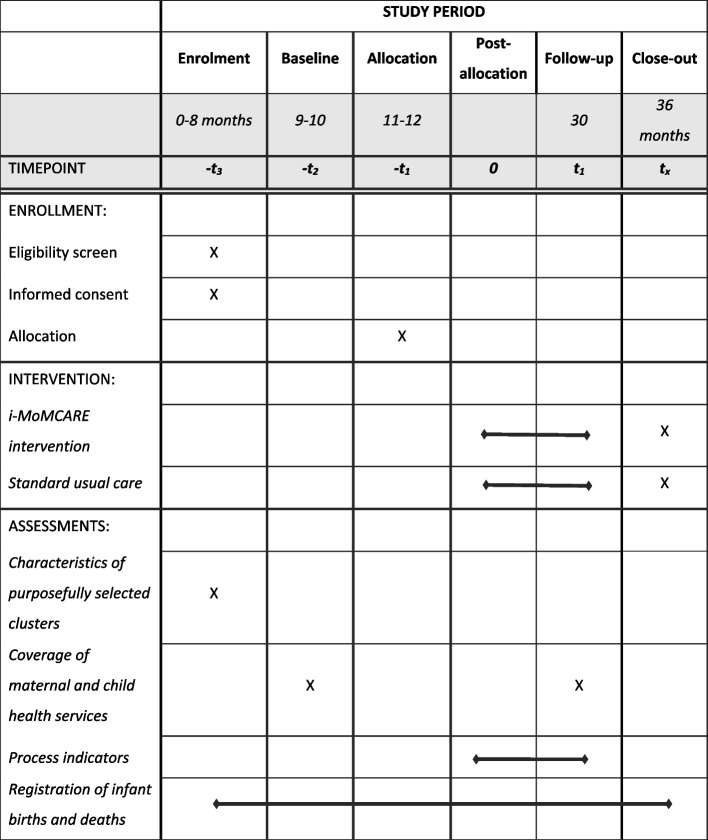
The Standard Protocol Items: Recommendations for Interventional Trials (SPIRIT) diagram

**Fig. 2:**
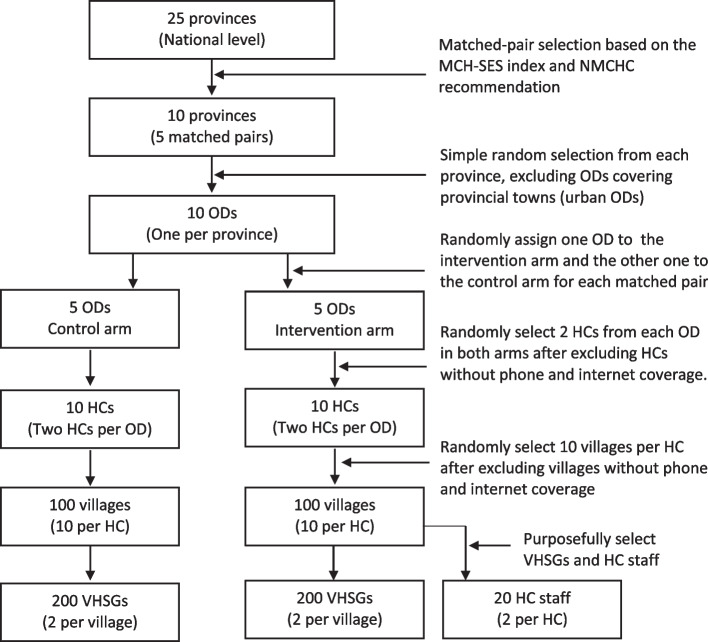
CONSORT study flow chart. HC, health center; MCH, maternal and child health; NMCHC, National Maternal and Child Health Center; OD, operational district; SES, socioeconomic survey; VHSG, village health support group

We will conduct this implementation research between 2022 and 2025 in 10 provinces. The study protocol adheres to the Standard Protocol Items: Recommendations for Interventional Trials (SPIRIT) diagram (Fig. 1), the Consolidated Standards of Reporting Trials (CONSORT) study flow chart (Fig. 2), and the SPIRIT Checklist (Additional file 1).

### Study design

This study is a two-arm stratified multi-stage, cluster randomized controlled trial (RCT). We will deploy digital health technology in five provinces. The five provinces have been selected based on the composite index of the MCH indicators and socioeconomic status (SES) indicators and consultation with the National Maternal and Child Health Center (NMCHC) of Cambodia’s MOH. We will conduct a literature review and gap analysis using in-depth interviews (IDIs), key informant interviews (KIIs), and focus group discussions (FGDs) with key stakeholders to acquire information necessary for introducing and adapting the digital health technology, successfully deployed in Gujarat, India, to the Cambodian rural settings.

### Randomization

The randomization will be done at the operational district level (cluster), the third tier of Cambodia’s health system. To address the issue of contamination that may arise and maintain comparability between the two arms (balanced randomization) at baseline, we performed a matched-pair selection of 10 provinces (five for each arm) out of the 25 provinces in Cambodia based on similar levels of MCH-SES index and NMCHC’s recommendations (Table 1). We used the principal component analysis approach [22] to construct the provinces’ MCH-SES index from a basket of indicators of ANC, delivery, and PNC from the Cambodia Demographic and Health Survey (CDHS) 2021 [4] and indicators of socioeconomic status from the National Population Census 2019 [23] and Cambodia Socioeconomic Survey 2019 (CSES) [24]. The indicators from CDHS 2021 included the proportion of pregnant women attending at least four ANC, the proportion of women delivering in a health facility, the proportion of women having a PNC visit in the first 2 days after delivery, the proportion of fully vaccinated (basic antigen) children aged 12–24 months, the proportion of fully vaccinated (according to the national schedule) among children aged 12–24 months, and proportion of children aged 12–24 months without vaccinations. The indicators from the population census 2019 included population density, literacy rate of the population aged 7 years and older, the proportion of the population aged 15 years and older with secondary education or higher, the proportion of households with electricity, and the proportion of households with piped water in a dwelling, while median monthly income per capita in thousand Khmer riels was from the CSES 2019.

**Table 1:** List of matched-pair provinces with their respective MCH-SES indices

Pair	Matched province	MCH-SES index	Matched provinces	MCH-SES index
1	Kampong Speu	1.11	Kampong Chhnang	1.38
2	Pailin	0.37	Pursat	0.12
3	Kampot	− 0.17	Preah Sihanouk	0.15
4	Oddar Meanchey	− 1.07	Kampong Thom	− 1.34
5	Preah Vihear	− 2.75	Ratanak Kiri	− 7.77

*MCH* Maternal and child health, *SES* Socioeconomic survey

As shown in Fig. 2, we will randomly select one operational district from each province after visiting study sites and excluding ODs in the provincial town (urban area) and those without Internet coverage. Within each pair of the 10 selected provinces, we will randomly assign one operational district to an intervention arm and the other to a control arm using Excel (Microsoft). Blinding to the participants, including HC staff, VHSGs, and the beneficiaries, would not be possible in this community-based intervention study. We also have no plan to make the allocation blind to the outcome assessors and data analysts. We will then randomly select two HCs from each operational district after excluding those covering less than 10 villages and without Internet coverage. In Cambodia, HCs provide a minimum package of activities, such as preventive and primary curative services, each serving approximately 10,000 to 20,000 people in villages under its coverage [25]. The number of HC staff may vary between eight and 11. The staff members typically include medical doctors (uncommon), midwives, nurses, and supporting staff.

### Sample size

We used a sample size calculator for the cluster RCT and a practitioner’s guide to sample size and power calculation by McConnell and Vera-Hernandez [26] to estimate sample size and power for this study. Given the absence of similar studies in Cambodia, our assumptions were based on those used in a similar study in India with some adjustments. We assumed the intra-cluster correlation (ICC) to be 0.02 and the effect size of 13%, slightly below the 15% in India [18]. We observed that the MCH coverage index constructed from indicators of at least four ANC visits, PNC visits in the first 2 days after delivery, and complete immunization of children aged 12–24 months in 2021 in our selected provinces is at 70%, higher than the 36% of the MCH composite index in India [27]. Our cluster size was approximately 200, assuming there are two HCs per operational district, 10 villages per HC, and 10 mothers or pregnant women per village. The confidence level was set at 95%, while each arm consisted of five clusters. After obtaining a sample of 1858 mothers (929 per arm) and a power of 85%, we accounted for a 5% non-response rate and arrived at a sample size of 1951 mothers for both arms.

### Main actors in the program

This trial will include three primary groups of participants. The first group will be the beneficiaries of the intervention, including pregnant women and mothers of infants and their newborn babies or children aged 0–24 months. The second group will be the *i-MoMCARE* facilitators (level 1 and 2 technology support personnel) assisting digital health technology users. The third group will include VHSGs and HC staff—the mobile application and web interface users.

#### i-MoMCARE facilitators

Two facilitators, the University of Health Sciences (UHS) staff, will provide technical support to the digital health technology users at either level 1 or level 2. The technical support at level 1 will include (i) collecting user requests and data, (ii) answering users’ phone calls (first contact), (iii) responding to users’ text messages, (iv) creating tickets for level 2 support, (v) providing features of the *i-MoMCARE*, and (vi) solving logistical and technical problems, such as password issues, menu navigation, verification of hardware and software, installation issues, and setup. The technical support at level 2 will involve more in-depth troubleshooting, including (i) using the facilitator dashboard available through the *i-MoMCARE* web interface for monitoring adherence to the *i-MoMCARE* intervention by using process indicators and undertaking housekeeping functions of the web interface, which includes dealing with the migration of pregnant women or mothers, duplication in registering cases, and verification of deaths reported by VHSGs and (ii) making occasional field visits to provide ongoing training, supervision, and encouragement to enhance motivation to poor performing VHSGs and sending a short message service (SMS) text to VHSGs and HC staff to inform them about high-risk cases diagnosed by VHSGs [18].

#### Village health support groups (VHSGs)

VHSGs are usually elected by their community members and based in the community under the HC’s coverage. VHSGs are community-based health volunteers with limited education; some do not even have education beyond primary school [28]. They will be the primary implementers of the *i-MoMCARE* intervention. They will communicate with the beneficiaries directly to narrow the gap in the knowledge of maternal, newborn, and child health and refer pregnant women and children to the nearest HC when necessary. One HC serves approximately 10 villages, and one village has one or two VHSGs serving 100 households or more. We will select 20 VHSGs (two VHSGs per village) for each HC in the intervention and control arms (200 from each arm). All VHSGs in the intervention and control arm will undergo MCH-related refresher training before the intervention. Only VHSGs in the intervention arm will be equipped with the mobile application.

#### Health center staff

We will invite 20 HC staff (two from each) working closely with VHSGs and involved with data management at the HC in the intervention arm to participate in the study. HCs usually provide minimum package activities, including ANC, deliveries, PNC, immunization for children and women, neonatal and child care, nutrition, communicable and non-communicable disease services, health education and promotion, and outreach activities [25]. VHSGs assigned to the intervention arm will conduct home visits and provide care, such as diagnosis, referral, counseling, and administering drugs, to mothers with complications who cannot visit the HCs. The HC staff will supervise and support VHSGs and verify VHSGs’ performance reports on such activities. One of the two selected HC staff for each HC will serve as a site coordinator providing feedback on VHSGs’ counseling and technical skills, organizing small group meetings with VHSGs, and facilitating the selection and replacement of VHSGs in the HC’s catchment areas. The site coordinators will further assist VHSGs who are poorly functional because of limited skills and knowledge, delays in payments, illness of family members, lack of family support, or other social barriers.

### Theory of change

**Fig. 3:**
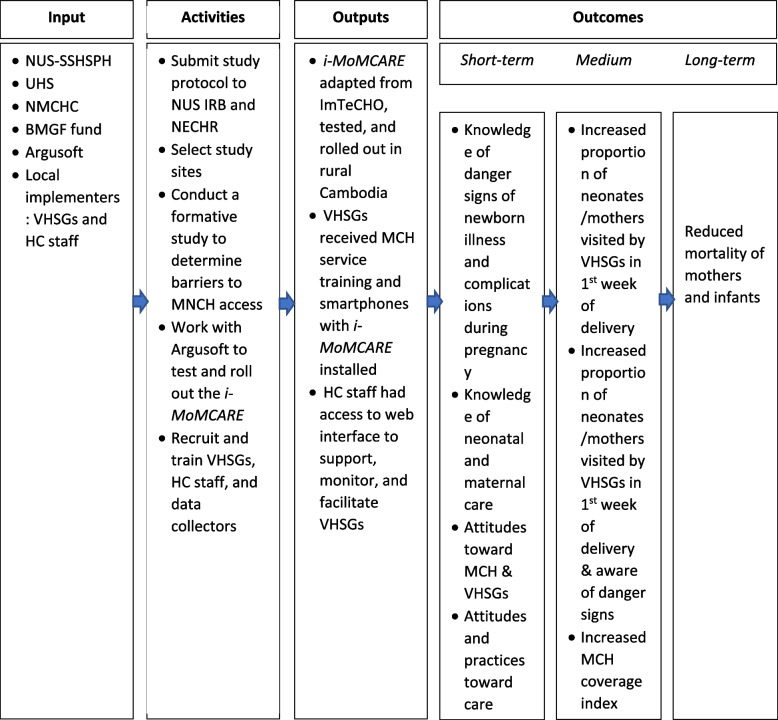
Logic model of the Innovative Mobile Technology for Maternal and Child Health Care (*i-MoMCARE*) intervention. BMGF, Bill & Melinda Gates Foundation; *i-MoMCARE*, Innovative Mobile Technology for Maternal and Child Health Care; ImTeCHO, Innovative Mobile-phone Technology for Community Health Operations; IRB, institutional review board; NECHR, National Ethics Committee for Health Research; NMCHC, National Maternal and Child Health Center; MCH, maternal and child health; NUS-SSHSPH, Saw Swee Hock School of Public Health of the National University of Singapore; UHS, University of Health Sciences; VHSG, village health volunteer group. The short-term outcomes are measured among mothers of children under 2 years or pregnant women, while the medium-term outcomes are measured among children under 2 years old

The logic model outlined in Fig. 3 describes how *i-MoMCARE* will attain its goal of reducing MMR and IMR in rural Cambodia. It comprises four major components—input, activities, outputs, and outcomes. Input refers to human and financial resources available for project implementation. The human resources will include faculty and researchers from the Saw Swee Hock School of Public Health of the National University of Singapore (NUS-SSHSPH), the University of Health Sciences (UHS), NMCHC, and Argusoft, an international technology service provider. The project receives funding from the Bill & Melinda Gates Foundation (BMGF). The activity column illustrates how we will pool human and financial resources to generate outputs presented in the output column. The activities include staff recruitment, study protocol submissions for ethical approval, qualitative formative studies, and collaboration with Argusoft and local implementers. We will translate the outputs into short-, medium-, and long-term outcomes.

We expect that *i-MoMCARE* will improve pregnant women’s knowledge about maternal and young infant care and attitudes toward using maternal, newborn, and child health services. As a result, it will enhance health-seeking behaviors and MCH outcomes indicated by the MCH composite coverage index illustrated in the subsection below. The ANC, PNC, and facility-based delivery rates are also expected to increase in the medium term. Achieving short- and medium-term outcomes would reduce MMR and IMR in the long term.

### Intervention

#### Phase 1: Single-arm pilot of i-MoMCARE

A single-arm pilot study will be conducted in the last quarter of year 1 to ascertain the reliability and success of its deployment. The initial stage will include three steps: (I) identifying gaps in MCH standard care, (II) identifying components of digital health for addressing the gaps in MCH standard care, and (III) piloting the operational delivery of the intervention.

##### Identifying gaps in MCH standard care

We will conduct a literature review and a consultation meeting with NMCHC. We will also conduct key informant interviews (KIIs) with stakeholders from NMCHC, NGOs (the World Health Organization [WHO] and United Nations Population Fund [UNFPA]), a provincial health department (PHD), and an operational district. KII topics will include digital health literacy, digital health policies, health technology assessment (HTA), and challenges and recommendations. We will conduct in-depth interviews (IDIs) with VHSGs and HC staff and focus group discussions (FGDs) with pregnant women and new mothers. IDI topics will include job scope, support, training, and barriers and challenges in their work. FGD topic guide will consist of questions about their experiences in accessing MCH services and interactions with VHSGs. Besides the KIIs with NMCHC and NGOs, we will interview stakeholders in the province where the pilot intervention will be implemented. An estimated number of KIIs, IDIs, and FGDs are presented in Table 2. The data collection will be carried out until information saturation is reached. We will include women who are pregnant at the time of recruitment, have the youngest child younger than 24 months old, have lived in the village in the past 6 months, have received pregnancy-related services from HCs, and have received ANC and PNC support from VHSGs.

**Table 2:** Number of qualitative interviews for gap analysis

Participant	KIIs	IDIs	FGDs
National maternal and child health center	1		
Non-governmental organizations	2		
Provincial health departments	1		
Operational districts	1		
Health center directors	3		
Health centers		6	
Village health volunteer groups		6	
Pregnant women and new mothers			3^a^
Total	8	12	3

*FGD* Focus group discussion, *IDI* In-depth interview, *KII* Key informant interview

^a^With 6–8 women in each group

##### Identifying technology components to address the gaps in standard care

We will identify technology components expected to tackle gaps in standard care based on consultations with Argusoft. A mobile application and web interface will be developed featuring functions that will assist the VHSGs in increasing MCH coverage and aid VHSGs and midwives in facilitating care for eligible mothers, newborns, infants, and children with complications. Various housekeeping functions, including troubleshooting, will be identified at this stage with support from Argusoft for optimal delivery.

##### Piloting operational delivery of i-MoMCARE

For piloting the *i-MoMCARE* implementation, we will select 50 VHSGs from five HCs (10 VHSGs per HC) and two HC staff under one selected operational district from one province. To avoid contamination, we will carry out the pilot in a province other than the provinces chosen for the intervention and control. The VHSGs and HC staff will be recruited for a 3-month pilot intervention and trained to use the mobile phone application and web interface. The pilot intervention will start with the preparation, training, and on-the-ground mentoring of VHSGs and HC staff on using the mobile phone application and a web interface for stabilizing and maintaining MCH service delivery. As shown in Table 3, we will assess the acceptability and feasibility of the pilot intervention through IDIs with VHSGs and HC staff who used the mobile application and web interface during the pilot. We will also conduct FGDs with six to eight pregnant women and mothers of children younger than 2 years old. Challenges and barriers related to the intervention delivery will be identified through the pilot, IDIs, and FGDs. Furthermore, we will finetune the mobile application and web interface to monitor implementation and progress in collaboration with Argusoft.

**Table 3:** Qualitative interviews after the pilot

Participant	In-depth interview	Focus group discussion
Health center staff	4	
Village health volunteer group	10	
Pregnant women and new mothers		2^a^
Total	14	2

^a^With 6–8 women in each group

#### Phase 2: Cluster randomized controlled trial

**Fig. 4:**
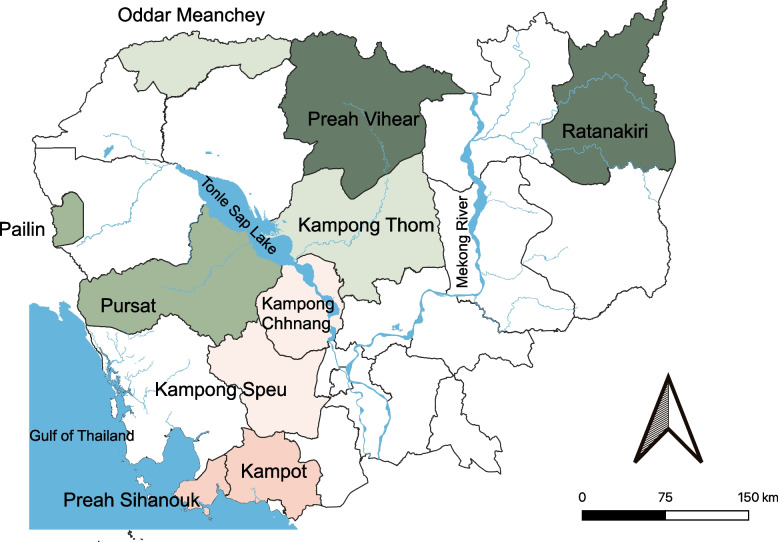
Map of the study sites (each color represents a paired province)

An open cluster randomized controlled trial will be conducted to evaluate the mobile application utilization and the intervention’s effectiveness in improving MCH service uptake and outcomes aided by the mobile application. We have estimated that approximately 200 VHSGs will be included in the intervention arm across five operational districts (ODs) in five provinces. Figure 2 shows a flowchart of VHSGs and the HC staff selection process for the intervention and control arms. Figure 4 illustrates the map of the 10 provinces proposed as study sites, five of which will be assigned to the intervention arm after randomly selecting one OD from each. We will randomly select two HCs covering approximately 10 villages from each OD. Two HC staff will be purposively selected from each HC in the intervention arm.

The designated HC staff will receive a web interface application as their job aid for supervising VHSGs, including real-time information about VHSGs’ performance in the form of process indicators and MCH service coverage. The web interface will provide timely information and tools for HC staff to facilitate the monitoring and supporting of VHSGs by supporting HC staff in their daily tasks, such as high-risk patient tracking, low-supply inventory alerts, supply chain management, electronic health records, vital events tracking, and automatic calculation of performance-based incentives and motivation for VHSGs. Two VHSGs from each of the 100 villages (a total of 200 VHSGs) will receive the mobile application connected to their respective HC in the intervention arm. The mobile application will provide job aid to VHSGs to enroll eligible women and children, schedule and review home visits, report outcomes of pregnancies, conduct home-based newborn care, follow-up visits of complicated cases, and report information about deaths, emergencies, migrations, referrals, and services, by logging into the application installed on the mobile devices every day except for Sundays. The mobile application will also help with behavioral change communication via brief educational videos about danger signs in pregnancies and newborns to be shown to pregnant women. At endline, we will conduct a qualitative acceptability and feasibility assessment of the intervention by conducting IDIs with VHSGs and HC staff from the intervention arm and FGDs with six to eight eligible pregnant women and mothers of under-two children in the five HCs selected for the IDIs. Approximate numbers of IDIs and FGDs are shown in Table 4. The interviews will be conducted until saturation of the information is reached.

**Table 4:** Qualitative interview at the endline

Participant	In-depth interview	Focus group discussion
Health center staff	10	
Village health volunteer group	10	
Pregnant women and new mothers		5^a^
Total	20	5

^a^With 6–8 women in each group

##### Control arm

As discussed in the study design, HCs in the control arm will be randomly selected from their ODs. The randomization process will be similar to those in the intervention arm. All villages under the HC’s catchment areas in the control arm will continue to receive MCH standard care provided by the government and NGOs and facilitated by VHSGs. VHSGs in the intervention and control arms will receive refresher training on maternal, neonatal, and childcare to avoid ethical issues that may arise and ensure that any change in outcomes in the intervention arm is due only to the *i-MoMCARE* intervention.

##### Criteria for discontinuing or modifying allocated interventions 

The intervention implemented in this study will be part of standard procedures endorsed by Cambodia’s national MCH program. We do not foresee discontinuation or modification of allocated interventions because the proposed activities will pose no more than minimal risk and not adversely affect participants’ welfare. Participation in the activities in the study will be voluntary, and the participants can withdraw from the study at any time.

##### Strategies to improve adherence to interventions

In this study, the implementing organizations will design and ensure that the intervention’s protocol is strictly followed. The medical procedures and laboratory tests conducted in the health facilities will not be part of this study and are in accordance with the national MCH guidelines. In addition, researchers from the National University of Singapore, which is not an implementing organization, will conduct regular supervision and interim analyses to ensure adherence to the study protocol.

##### Relevant concomitant care permitted or prohibited during the trial 

Due to the trial’s nature, no concomitant care would be specifically permitted or prohibited during the study. We will conduct the community-based intervention according to standard operating procedures in a “real-world” setting.

##### Provisions for post-trial care

Post-trial care will be provided in accordance with the standard care as defined in the national MCH guidelines under the purview of the NMCHC.

### Outcomes and measures

We have selected primary and secondary outcomes based on the study in Gujarat, India [18]; Cambodia’s National Guideline for Implementing the Service Package of Antenatal Care, Delivery, and Postnatal Care 2019 [29]; and consultations with the NMCHC. We have also selected other primary outcomes, such as the proportion of neonates/mothers visited at home by VHSGs within the first week of delivery and mothers receiving information regarding the danger signs of newborn illnesses, based on a previous formative study [30].

#### Primary outcomes

VHSGs will facilitate the primary outcomes of interest throughout the continuum of care, including (1) the proportion of neonates/mothers visited at home by VHSGs at least twice within the first week of delivery, (2) the proportion of mothers receiving information from VHSGs regarding danger signs of newborn illnesses within the first week of delivery, and (3) the MNCH composite coverage index, consisting of four dimensions, namely maternal care, neonatal care, infant care, young childcare, and care-seeking when having complications.

The MCH composite index was initially introduced by Boerma et al. [31, 32]. It captures a complete continuum of care of mothers and children by including four dimensions: family planning, maternal and newborn care, immunization, and treatment of children. Following Modi et al. [27], we will exclude family planning to focus more on the role of the VHSGs in evaluating the effectiveness of mobile health technology. The three components will be equally weighted. The index will be calculated using the following formula: MCH-CI = 1/3 (¼ APNC + ½ IMMU + 1/3 TREAT), where APNC denotes antenatal and postnatal care, IMMU denotes immunization among children, and TREAT denotes the treatment of children. The index will vary between 0 and 100, with a higher index score indicating a higher MCH coverage. We will use the MCH coverage index to evaluate the effectiveness of the intervention. We have conjectured that the coverage rate should be well above 60% to be meaningful since earlier estimates of the compelling national coverage of maternal health services (ANC and facility delivery) and sick childcare (diarrhea, pneumonia, and fever) in 2014 were 56.4% and 59.1%, respectively [33]. Another recent study using a slightly deviated measure of the mother and infant care continuum shows that the national coverage was 49.4% [34].

APNC will consist of four sub-components: (1) the proportion of mothers visited by VHSGs at least three times in the last pregnancy, (2) the proportion of mothers with at least four ANC visits in the last pregnancy and one tetanus vaccine injection, (3) the proportion of mothers delivered at a health facility (public or private), and (4) the proportion of mothers with at least three PNC visits in the last pregnancy. IMMU will consist of two sub-components: (1) the proportion of young infants aged 12–24 months fully vaccinated based on the national guideline with one dose of Bacillus Calmette–Guérin (BCG) vaccine for tuberculosis; three doses of polio vaccine given as oral polio vaccine (OPV), inactivated polio vaccine (IPV), or combination of OPV and IPV; three doses of diphtheria, pertussis, and tetanus (DPT) vaccine; and one dose of measles vaccine given as measles-rubella (MR) and (2) the proportion of young infants exclusively breastfed in the first 6 months. TREAT will consist of three sub-components: (1) the proportion of neonates with complications in the first month of the last delivery who seek care from VHSGs, (2) the proportion of infants aged two to 24 months with diarrhea in the last 2 weeks and receiving oral rehydration therapy (ORT) from VHSGs, and (3) the proportion of infants aged 2 to 24 months with acute respiratory infections in the last 2 weeks and seeking care from VHSGs.

#### Secondary outcomes

As shown in Table 5, the secondary outcomes of interest will include MCH service coverage and process indicators of the *i-MoMCARE* intervention, adapted from previous studies [18, 29, 35]. We will measure these indicators to further determine the degree of effectiveness of the intervention.

**Table 5:** List of secondary outcomes of interest in the *i-MoMCARE* trial

**Outcomes indicators and description**
**Maternal outcomes**
*Antenatal care (ANC)*
1. The proportion of mothers completing the first ANC visit timely and those having at least four ANC visits
2. The proportion of mothers receiving recommended supplements, HIV and syphilis tests, and immunization during ANC visits
3. The proportion of mothers visited at home or called by VHSGs at least three times during the pregnancy
4. The proportion of mothers with complications during their last pregnancy and seeking care from health providers or VHSGs
*Delivery*
1. The proportion of mothers delivered by a skilled provider and in a health facility
2. The proportion of mothers staying at least 3 days at a health facility after delivery for post-delivery care
3. The proportion of mothers delivering at less than 37 weeks of pregnancy (pre-term delivery)
4. The proportion of mothers delivering low-birth-weight babies (< 2.5 kg)
5. The proportion of mothers experiencing complicated delivery
*Postnatal care (PNC)*
1. The proportion of mothers with a postnatal checkup during the first 7 days after delivery by skilled health providers
2. The proportion of mothers with at least three PNC visits by the 23rd month of delivery
3. The proportion of mothers receiving counseling about body and hand hygiene, nutrition for the mother, breastfeeding, and risk symptoms for the mother and newborn during PNC visits
4. The proportion of mothers with complications within the first month of the last delivery and seeking care from skilled health providers or VHSGs
*Neonatal (0–28 days) outcomes*
1. The proportion of neonates breastfed within the first hour of birth
2. The proportion of neonates examined with the risk symptoms
3. The proportion of neonates visited and satisfactorily examined by VHSGs during their home visits after delivery
4. The proportion of mothers receiving satisfactory counseling about neonatal care from skilled health providers after delivery
5. The proportion of neonates with complications within the first month of the last delivery and seeking care from skilled health providers or VHSGs
*Young infant (2–12 months) outcomes*
1. The proportion of mothers exclusively (≥ 6 months) breastfeeding their child
2. The proportion of young infants receiving recommended vitamin supplements
3. The proportion of young infants fully vaccinated with basic antigens^a^
4. The proportion of young infants with severe diarrhea and receiving oral rehydration therapy from health providers or VHSGs within the last 2 weeks
5. The proportion of young infants with acute respiratory infections/fever and receiving care from health providers or VHSGs within the last 2 weeks
6. The proportion of young infants receiving growth monitoring checkups and follow-ups
*Young child (13–24 months) outcomes*
1. The proportion of mothers continuously (beyond 6 months) breastfeeding their child
2. The proportion of mothers receiving birth-spacing advice from skilled health providers
3. The proportion of young children fully vaccinated according to the national schedule^b^
4. The proportion of stunted, wasted, and underweighted children (under 2 years) (will be captured by anthropometric measures)
*Process indicators*
1. The proportion of days VHSGs and HC staff logging in to *i-MoMCARE* mobile phone and web-based application, respectively (login rate)
2. The proportion of scheduled tasks completed by VHSGs and HC staff in *i-MoMCARE* mobile phone and web-based application, respectively (task completion rate)
3. Number of pregnancies and births registered using mobile phones against the total number of pregnancies and births
4. Number of complicated maternal, newborn, and child cases identified against expected
5. Stock-out rate (proportion of times when a drug or equipment is unavailable when required, e.g., non-availability of antibiotics in case of child pneumonia)

*ANC* Antenatal care, *HC* Health center, *PNC* Postnatal care, *VHSG* Village health support groups

^a^A young infant aged 2–12 months is considered fully vaccinated if the infant has received the BCG vaccine, three doses of polio and DTP vaccines, and a single dose of measles vaccine [4]

^b^A young child aged 12–24 months is considered fully vaccinated according to the national schedule if the child has received all basic antigens and a birth dose of hepatitis B vaccine, a dose of IPV, three doses of hepatitis B and Hemophilus influenza type B vaccine (given as part of DPT vaccine), and three doses of the pneumococcal vaccine [4]

### Data collection and management

Data collection, entry, quality assurance, monitoring, and security maintenance will be supervised and coordinated by UHS’s Public Health Department, the lead implementer of the *i-MoMCARE* program. The Saw Swee Hock School of Public Health of the National University of Singapore (NUS-SSHSPH) will provide technical support to Cambodia-based implementing partners.

#### Quantitative data

##### Mobile and web-based applications

The process indicators will be collected from the web interface of the *i-MoMCARE* program during the intervention. Data from the mobile application will be linked to the web interface and stored in encrypted cloud storage hosted by the UHS. Given the high sensitivity of personal health data, we will develop a data management strategy for the data collected by the mobile application and web interface to ensure the data security and privacy and confidentiality of the participants. Data will be backed up regularly into encrypted computers. Only research team members will have access to the data.

##### Household survey and anthropometry measures

We will conduct household surveys at baseline and endline to measure the primary and secondary outcomes of interest in control and intervention arms. We have developed a household survey questionnaire (Additional file 2) based on the tools used in the Cambodia Demographic Health Survey (CDHS) 2021 and the National Guidelines for Implementing the Service Package of Antenatal, Delivery, and Postnatal Care [4, 29]. We will pre-test the tools before the data collection. Mothers of children aged 6–24 months and pregnant women aged 18–49 years will be eligible to participate in the surveys. The survey will collect household demographic information, durable assets, access to ANC, delivery, and PNC services, health insurance coverage, social health protection, health expenditures, and child anthropometric information. We will use an online platform to store the data and an electronic tablet installed with the data collection application equipped with Internet access to enter the data by trained interviewers. A village-level household population list from the population census in 2019 will be used as a sampling frame for household selection.

We will measure weight and height and check for edema of the eligible children (aged 6–24 months) to measure their nutritional status during the household survey. Weight will be measured in kilograms (kg) using the United Nations Children’s Fund (UNICEF) Uniscale with a precision of 100 g (one decimal point). Height will be measured using a length board from UNICEF in centimeters with a precision of one millimeter. The enumerators will measure height and weight twice. A research team member with a child nutrition background will train the interviewers to perform the anthropometric measurements. All enumerators will practice the anthropometric measurements during the pre-test. They will be instructed to calibrate the weighing scale before collecting data.

We will conduct data quality assurance exercises during and after data collection. The research team will review stored and regularly updated data on the server by synchronizing them with electronic tablets. Questions arising from the reviews will be directed to the data collection team leaders. After completing the data collection, the team will check the data set again for inconsistency, outliers, irrational magnitude, errors, and illogical skipped patterns in collaboration with the data collection team leaders. We will store the cleaned data in a desktop computer with a password at UHS’s Department of Public Health office.

#### Qualitative data

We will conduct KIIs, IDIs, and FGDs with the community and stakeholders recruited using the purposive sampling method at three study stages—gap analysis, pilot, and intervention. Tables 2, 3, and 4 show each stage’s estimated number of interviews. We will train the enumerators to conduct the interviews and write the discussion and field notes. We have developed the topic guides in consultation with the UHS and NMCHC. Interviews will be conducted face-to-face in Khmer and recorded using an audio recorder. The research team will transcribe the Khmer transcriptions and translate them into English for thematic analyses, save the recorded audio and transcriptions/translations on a password-protected computer, and keep the field discussion and notes in a locked cabinet in the study team’s office. We will assign unique identification numbers instead of participants’ identifiers to protect their privacy and confidentiality. Additional file 3 presents qualitative data collection tools.

### Analysis plan

#### Quantitative data

##### Data from household surveys, mobile applications, and web interface

We will use Stata 17 (StataCorp LLC, Lakeway Drive) for quantitative data analyses. Since the randomization will be performed at the cluster level, all pregnant women and infants in the intervention arm will be eligible for the program. We will conduct intention-to-treat (ITT) analyses to examine the *i-MoMCARE*’s impact on outcomes of interest. Additional analyses will be per the study protocol analyses. We will analyze household data by considering cluster randomization design. The matched-pair method introduced in the earlier section is to achieve balanced randomization rather than representing a matched dataset when individual participants serve as their controls. Therefore, our analysis must consider this matching. We will test (i.e., Student’s *t*-test and chi-square test) the differences in the means or proportions of the maternal and child characteristics in the intervention and control arms at the beneficiary and cluster levels to observe balances in the study arms at baseline. In case of an imbalance in certain specific characteristics, we will apply a generalized estimating equation (GEE) approach to account for this imbalance. We will employ a difference-in-difference method using the GEE approach for all outcomes of mothers, pregnant women, and infants collected at baseline and endline. We will cluster standard errors at the village level to account for intra-cluster correlation in the characteristics of mothers. To account for multiplicity that may arise from testing multiple endpoints (i.e., primary and secondary outcomes) and lead to false rejection of the null hypothesis of no effect (type I error), we will apply the Bonferroni method and resampling-based method (i.e., bootstrapping) to obtain appropriate *p*-values [36].

To calculate children’s nutritional status, we will enter data on weight, height, sex, and date of birth into the Essential Nutrition Action software (ENA 2011) to compare the weight-for-age, height-for-age, and weight-for-height data to the international WHO reference standards. Data from the mobile phone application and web interface will be analyzed using a before-and-after method to determine the changes in the process indicators (Table 5).

We will conduct a sensitivity analysis to ascertain the effect size in our analyses. The GEE method has recently been criticized for inflating the type 1 error, resulting primarily from the incorrect standard errors obtained from the GEE method when the number of clusters is small [37]. Since the number of clusters in our study will be 10 ODs (< 30 clusters), we will conduct further analyses using the finite-sample corrected GEE bias correction [38, 39] and covariate adjustment methods [40, 41] to obtain the correct standard errors and make appropriate inferential analyses.

##### Analysis methods to handle protocol nonadherence and missing data 

We will adopt intention-to-treat analyses and imputation methods for missing data.

##### Interim analyses

A data analyst masked to intervention allocation will conduct interim analyses.

#### Qualitative data

We will use NVivo 12 (QSR International) for qualitative data analyses. Data from KIIs, IDIs, and FGDs will be analyzed thematically using both inductive and deductive coding [42]. We will adhere to the six phases of reflexive thematic analysis outlined by Braun and Clarke [43–45]. Preceding the analysis, the transcribed and translated data will be de-identified and rigorously cleaned for translation accuracy, a process verified by bilingual study team members using the original interview audio recordings. Beginning with the first phase, we will immerse ourselves in the data by thorough and repeated readings. Moving to the second phase, the team will tag pertinent features related to our research questions, assigning code labels. The codebook will take shape through an inductive and deductive approach, informed by topic guides and research questions, and coders will apply it to all transcripts, introducing and discussing new codes as needed. For the third phase, we will review and cluster similar codes to uncover patterns of shared meaning, potentially employing visual tools for organization. Subsequently, in the fourth phase, we will systematically review and discuss these patterns for each data segment and the dataset as a whole. In the fifth phase, we will refine emerging themes through thorough discussion, ensuring precise definition and nomenclature. Finally, in the last phase, the analysis will culminate in comprehensive written documentation of the final analysis findings.

#### Cost-effectiveness analysis

We will perform an economic evaluation following the principles, methodological specifications, and reporting standards recommended by the *International Decision Support Initiative Reference Case for Economic Evaluation (iDSI Reference Case)* [46]. Health benefits and costs will be measured from the societal perspective to consider the impact on the program, health system, and patient levels. Using a bottom-up costing approach, we will estimate the recurrent and fixed costs, including manpower (e.g., staff salaries), capital (e.g., equipment), other operating costs (e.g., consumables), and overheads. Considering the depreciation and opportunity costs, capital costs will be annualized over the product’s expected lifespan.

Program costs will include costs related to the intervention development, such as VHSG training and software development, based on the financial records of the implementing teams (UHS and NUS-SSHSPH). Research costs will be recorded but not included in the analyses as it has no implications on adopting the intervention in the real world. Health system costs will consist of the costs of providing preventive and curative MCH services at public health facilities based on WHO-CHOICE estimates and verified by PHDs. We will include items not included in the program but likely required if the intervention is adopted by the health system, such as the costs of integrating the intervention into the health system. The costs of digital integration will be based on consultations with the technology provider (Argusoft) and government officials with knowledge of digital infrastructure. We will consider the existing digital infrastructure required to support the intervention sunk-in costs and exclude them from the analyses.

We will estimate patient costs based on out-of-pocket expenditures reported by patients in the household surveys, including health and non-health costs for treatment of pregnancy complications and newborn and infant illnesses, ANC, delivery, and PNC in the public health system. Costing will only include expenditures incurred in the public health system to understand cost implications on the national healthcare budget. We will use data on service utilization from the household surveys and the registration information collected by the surveillance team, birth and death registration record, and, where possible, aggregate data provided by NMCHC in addition to public health system costs and out-of-pocket expenditures to estimate a standardized unit cost of delivering preventive and curative MCH services in the intervention and control arms.

We will conduct the economic evaluation using a cost-effectiveness analysis (CEA) by estimating the incremental costs and benefits of the intervention compared to the control, summarized in the incremental cost-effectiveness ratio (ICER). Decision-analytic modeling will be performed to estimate the cost per infant and maternal death averted, years of life saved (YLS) by averting an infant or maternal death, illness episode prevented, and disability-adjusted life years (DALY) averted between intervention and control. The incremental cost is the cost of integrating and using the intervention in the public health care system and the additional utilization of MCH services arising from the intervention.

The modeling will be based on improving service utilization, thereby reducing illnesses or complications during pregnancy and after birth and reducing maternal, neonatal, and child mortality. The reduction in mortality leads to a reduction in the years of life lost (YLL) due to premature mortality and a decrease in DALY. We will use disability weights in the Global Burden of Disease 2019 to compute years lived with disability (YLD) due to maternal complications and neonatal and infant illnesses. We will develop the model based on the evidence of preventive and curative services shown in the literature [47, 48] and the LiST (Lives Saved Tool) model [49]. The modeling will explore different time horizons, factoring in the downstream costs and benefits at varying intervention effects and when scaled up to more provinces and the national level.

We will employ a 3% discount rate for both costs and benefits, and sensitivity analyses will be done using different discount rates to account for uncertainty. In the absence of a local cost-effectiveness threshold (CET) for Cambodia, a less than one-time gross domestic product (GDP) per capita will be considered as “very cost-effective,” and between one to three times of GDP per capita will be regarded as “cost-effective” [50]. We will adapt the Consolidated Health Economic Evaluation Reporting Standards (CHEERS) checklist to report the cost-effectiveness outcomes. We will capture the aforementioned economic plans in a health economic analysis plan in consultation with the NMCHC and other stakeholders, such as WHO, through KIIs with personnel with expertise or knowledge in health financing or health technology assessments (HTA). The KII will cover the HTA process and requirements, including the goals and types of economic evaluations. As Cambodia does not have a national HTA organization or formal HTA policies and procedures, the consultation and KIIs will engage MOH in the financial evaluation process and, more broadly, about HTA. The KIIs will be conducted in phase 1 as part of the gap analysis.

Through the KIIs, we will engage with the MOH to share and engage government stakeholders in the economic evaluation. The KIIs will be conducted at baseline and endline as part of the qualitative evaluation. At the baseline, one KII will be conducted with regional partners (WHO and UNFPA) to understand best practices for HTA, and another KII will be done with NMCHC to understand if there is any HTA process and requirements in Cambodia, including preferred approaches for HTA. The interview data will help refine the economic evaluation methodology. At the endline, we will conduct a sharing session with NMCHC and other stakeholders to share the study findings and hear their views on how economic evaluation can feed into policymaking and shape the process for HTA establishment in Cambodia.

### Oversight and monitoring

#### Composition of the coordinating center and trial steering committee

The coordinating center will be based at UHS, the primary implementing organization. The steering committee will comprise representatives of NMCHC, NUS-SSHSPH, UHS, and key stakeholders working on MCH in Cambodia (e.g., WHO, UNICEF, UNFPA, USAID, and local NGOs). Data will be stored and managed by the team at UHS, with technical support from NUS-SSHSPH and Argusoft. The research team from NUS-SSHSPH will be responsible for data analyses. The steering committee will adjudicate the endpoints and develop the findings dissemination plan in consultation with the data monitoring committee. During the trial preparation phase, the steering committee will meet every month, and during the intervention, the committee will meet every quarter (or ad hoc meeting). The research team will produce meeting reports to update research progress or report any issues during the project implementation.

#### Composition of the data monitoring committee and the reporting structure

The data monitoring committee (DMC) will comprise Argusoft, NUS, and UHS representatives. We will form the DMC independently from the funders. The committee members are not directly involved in the project; however, they are affiliated with organizations within the project consortium (UHS and NUS) and the technical support firm (Argusoft). The DMC will be responsible for independently assessing the integrity and validity of the RCT. We will present the interim analyses to the DMC. The DMC will report the trial progress and results to the steering committee. The DMC will meet twice a year or as needed.

#### Adverse event reporting and harms

The study will impose no major perceivable risks, and the potential psychological distress in the interviews will be minimal. Participation is voluntary, and the decision to decline or discontinue this study at any time during the study will not have any negative consequences. All participants and those who refuse to participate in the survey will receive standard MCH care.

#### Plans for auditing trial conduct

We plan to conduct at least four monitoring visits to each operational district during implementation. Field supervisors will conduct at least eight monitoring visits (e.g., every quarter). During monitoring visits, we will review consent forms, completeness of the data collection forms, compliance with the trial protocol, and follow up on any anomalies in recruitment and data. The research team will monitor data, recruitment processes, and interim results and report to the DMC.

#### Plans for communicating significant protocol amendments

We will communicate amendments to the protocol to all relevant parties, including trial participants and ethical committees, the NMCHC and all partners, investigators, ethics boards in Cambodia and Singapore, the trial registry, and journals.

#### Dissemination plans

We will share the study findings in dissemination workshops with key stakeholders and in peer-reviewed journals. Following the International Committee of Medical Journal Editors guidelines, we will determine authorship credit based on the research team members’ contribution to the conception, design, execution, or analysis and interpretation of the data. All authors should be involved in drafting the article or revising it critically for important intellectual content and must have read and approved the final version of the manuscript. We will publish information from the full protocol and tools in a peer-reviewed journal. The relevant data analyzed during the development of this study protocol are available upon request from the corresponding author.

##### Ethical considerations

The trial will follow the Good Clinical Practice guidelines [51]. We will amend and resubmit the study protocol to the aforementioned ethical committees if there are significant changes. At the beginning of each interview, participants will be informed about the study’s objectives, data confidentiality, the risks and benefits of their participation, the voluntary nature of the participation, and their rights to refuse or discontinue the study at any time. Additional file 4 provides patient information sheets and consent forms. Interviews will be conducted in settings that protect the participants’ privacy, and we will use study identification numbers instead of personal identifiers. Only the project staff from NUS-SSHSPH and UHS will have access to the personal data in the trial. We will not publish the data and discard them after disseminating the results.

### Patient and public involvement

We commit to fostering a collaborative and inclusive approach to maximize the impact of our intervention by actively involving beneficiaries and stakeholders. We have actively engaged with representatives of women of reproductive age at the formative study sites and stakeholders involved in MCH across multiple levels, including NMCHC, PHDs, ODs, HCs, VHSGs, and NGOs, to conduct a comprehensive gap analysis and develop protocols and tools. This engagement remains a priority as we progress through the study’s implementation, evaluation, and dissemination phases. To ensure their ongoing involvement, we will extend invitations to representatives from each stakeholder group to join a project steering committee. This committee will be pivotal in guiding the study’s direction and decision-making. Meetings of the project steering committee will convene every 3 months to assess project progress, gather feedback, identify potential issues, and advise on any challenges that may arise throughout the project’s lifecycle.

### Anticipated risks and benefits to human subjects

There are no significant perceivable risks to participating in this study, aside from the inconvenience of taking the time to answer questions for the beneficiaries and the time to attend the *i-MoMCARE* mobile application and web interface training course for VHSGs and HC staff. Additionally, we foresee the potential psychological distress to be minimal. There are no direct benefits for the participants; however, this study will contribute to integrating digital health technology to bridge gaps and ultimately improve MCH services in rural Cambodia. However, we anticipate several potential risks that we can address. For instance, if the respondents incur any morbidities during the interview, they must be accompanied to the nearest health facility, whether in the intervention or control arm. Besides, if domestic violence occurs during the data collection, the interviewers will stop the interviews and call local authorities, such as the village chief, to resolve the problem. In addition, VHSGs will observe signs of aggression or violence in the participants to avoid potential harm.

### Compensation and incentives

The VHSGs and HC staff in the intervention arm will receive a monthly stipend of USD 15 and USD 20 during the pilot and the intervention period. Participants in IDIs, KIIs, FGDs, and household surveys will receive USD 2.5 for each interview.

## Discussion

Improving MCH services uptake and maternal, neonatal, and infant health are public health priorities in the Sustainable Development Goals [52]. Although the overall MMR and IMR in Cambodia have drastically declined over the last two decades, the rates among people living in rural areas remain disturbingly high [1]. As seen in other LMICs, urban versus rural disparities in MCH services access and uptake remain large in Cambodia. Mobile technology has been used to improve community-based health interventions to bridge the gaps [53, 54], along with adopting digital health innovations in solving other public health problems in the country [55].

Evidence from Modi and colleagues [18] suggests mHealth technology’s success in improving MCH services coverage and quality in hard-to-reach settings in rural Gujarat, India [18]. By fully adopting the technology, this study aims to test and implement mobile phone- and web-based applications in a rural Cambodian context using a cluster randomized controlled trial. Findings from this study will drive public health policy changes in Cambodia, while lessons learned may benefit other LMICs where digital health interventions can be advantageous. This study has come at the right time when Cambodia’s MOH has committed to developing digital health innovations in the country. The government has established the National Committee for Digital Health and is now forming the Department of Digital Health under the MOH.

### Strengths and limitations

A considerable strength of this study is the cluster randomized controlled trial design using the innovative digital health technology successfully implemented and evaluated in the tribal communities in India [56]. This application does not require technology integration into the national platform, as this study will fully adapt this innovative approach. Additionally, the population under the technology coverage is substantially smaller than that in India, suggesting no critical challenges to this application. On top of that, the implementation will be supported by the national program, which spearheads the nationwide implementation of MCH service provision.

Despite these strengths, this study has several technical and operational challenges and limitations. On the technical side, a low level of education and digital literacy among VHSGs could be a challenge, while different mobile network providers in other geographical locations are another. On the operational side, we anticipate a high turnover rate among VHSGs due to the voluntary nature of their work, the demand for household chores, and other competing job opportunities in the community. The local partners—UHS and NMCHC—will address these challenges by ensuring the minimum level of education of nine years, selecting mobile network providers based on their geographical presence, and providing constant motivation and support to VHSGs.

## Conclusion

This cluster randomized controlled trial aims to develop, implement, and evaluate the applicability and efficacy of the *i-MoMCARE* intervention to support VHSGs and HC staff in rural communities to increase the coverage of and access to MCH services in Cambodia. This trial is the first study using digital health technology to support community-based health volunteers providing MCH services in Cambodia. It will contribute to advancing digital health use in primary healthcare interventions, which remain in their infancy in LMICs. This study will contribute to Cambodia Digital Government Policy 2022–2035 in general and the HealthTeach Roadmap in particular. Lessons learned from this trial can inform digital health interventions for improving MCH in other LMICs.

## Trial status

Protocol version 1, April 7, 2023. The recruitment for intervention is expected to commence in June 2023 and will continue until May 2025.

### Supplementary Information

**Additional file 1.** SPIRIT Checklist.

**Additional file 2.** Quantitative tools.

**Additional file 3.** Qualitative tools.

**Additional file 4.** PIS & informed consent forms.

## Data Availability

Not applicable.

## Abbreviations

ANC: Antenatal care
BMGF: Bill & Melinda Gates Foundation
CDHS: Cambodia Demographic and Health Survey
CEA: Cost-effectiveness analysis
CET: Cost-effectiveness threshold
CHEERS: Consolidated Health Economic Evaluation Reporting Standards
CONSORT: Consolidated Standards of Reporting Trials
CSES: Cambodia Socioeconomic Survey
DAC: Design, Analyze, and Communicate
DALY: Disability-adjusted life years
DPT: Diphtheria, pertussis, and tetanus
FGD: Focus group discussions
GDP: Gross domestic product
GEE: Generalized estimating equation
HC: Health center
ICC: Intra-cluster correlation
ICER: Incremental cost-effectiveness ratio
IDI: In-depth interviews
IMR: Infant mortality rate
i-MOMCARE: Innovative Mobile Technology for Maternal and Child Health Care
ImTeCHO: Innovative Mobile-phone Technology for Community Health Operations
IPV: Inactivated polio vaccine
ITT: Intention-to-treat
LMIC: Low- and middle-income countries
MCH: Maternal and child health
MMR: Maternal mortality ratio
MOH: Ministry of Health
NECHR: National Ethics Committee for Health Research
NMCHC: National Maternal and Child Health Center
NUS-IRB: National University of Singapore’s Institutional Review Board
NUS-SSHSPH: Saw Swee Hock School of Public Health of the National University of Singapore
OD: Operational district
OPV: Oral polio vaccine
PHD: Provincial health department
PNC: Postnatal care
RCT: Randomized controlled trial
SES: Socioeconomic status
SPIRIT: Standard Protocol Items: Recommendations for Interventional Trials
UHC: Universal health coverage
UHS: University of Health Sciences
UNFPA: United Nations Population Fund
UNICEF: United Nations Children’s Fund
VHSGs: Village health support groups
WHO: World Health Organization
YLD: Years lived with disability
YLL: Years of life lost
YLS: Years of life saved
