# Supplementary material for: i-MoMCARE: Innovative Mobile Technology for Maternal and Child Health Care in Cambodia—study protocol of a cluster randomized controlled trial
Source: Trials. 2023 Oct 26;24:692. doi: 10.1186/s13063-023-07724-z (PMC10601211; doi:10.1186/s13063-023-07724-z)
Supplement: Supplementary file 2 — Additional file 2. Quantitative tools. [file 13063_2023_7724_MOESM2_ESM.docx]

**Household Survey Questionnaire (Baseline, Midline, and Endline)**

**Project title:** *i-MoMCARE* – Innovative Mobile Technology for Maternal and Child Health Care in Cambodia: study protocol of a cluster randomized controlled trial

**Identifier**:

HH ID: **__ __ __ __** Interviewer ID: **__ __** Date of the interview: **__/__/____**

| **Geographical location**:  Province name:__________  Province ID: _____________  Operational district name: ______________  Operational district ID: _________________  Administrative district name:______________ | Administrative district ID:______________  Commune name:____________  Commune ID:____________  Village name:___________  Village ID:_____________  Health center name:__________  Health center ID: _____________ |
| --- | --- |

**Eligibility screening**

|  | 1^st^  attempt | 1^st^  appointment | 2^nd^  attempt | 2^nd^  appointment | 3^rd^  attempt | 3^rd^  appointment |
| --- | --- | --- | --- | --- | --- | --- |
| Date | / /20-- | / /20-- | / /20-- | / /20-- | / /20-- | / /20-- |
| Time |  |  |  |  |  |  |
| Interviewer ID |  |  |  |  |  |  |
| 0.1 | Does your household have a child/children aged from zero to 24 months living in your household? | | No | | 0 | |
|  |  |  | Yes | | 1 | |
| 0.2 | Is the mother of the child living in your household? | | No | | 0 | |
|  |  |  | Yes | | 1 | |
| 0.3 | Have you lived in this village/commune in the past 6 months? | | No | | 0 | |
|  |  | | Yes | | 1 | |

**1. Start of interview**

| The enumerator can start the interview only after receiving informed consent from the targeted mother. | | | |
| --- | --- | --- | --- |
| 1.1 | Are you a mother of a child/children aged less than 24 months in this household?  [IF THE RESPONSE IS A “NO”, THE INTERVIEWER HAS TO RESCHEDULE THE APPOINTMENT OF THE MOTHER LIVING IN THIS HOUSEHOLD.] | No | 0 |
|  |  | Yes | 1 |

**2. Sociodemographic and economic characteristics**

*Mother’s questions*

| No | 2.1 What year were you born?  [YEAR] | 2.2 How old are you? | 2.3 What is your marital status?  [CODES BELOW] | 2.4What is your highest level of education completed?  [CODES BELOW] | 2.5 What is your occupation?  [CODES BELOW] | 2.6 How many members are in your household? | 2.7 How long have you lived in this commune?  [YEAR] |
| --- | --- | --- | --- | --- | --- | --- | --- |
| 1 |  |  |  |  |  |  |  |
| 2 |  |  |  |  |  |  |  |

***Codes for 2.3****: 1. Never married, 2. Married, 3. Never married but living together, 4. Divorced / Separated, 96. Other (Specify__________)*

***Codes for 2.4****: 0. No education, 1. Primary school 2. Secondary school 3. High school 4. Higher*

***Code for 2.5:*** *0.* *Unemployed, 1. Agricultural sector 2. Unskilled manual work 3. Skilled manual work 4. Sales and services 5. Clerical 6. Professional/technical/ managerial*

| **N** | **Question** | **Answer** | **Code** |
| --- | --- | --- | --- |
| 2.7 | What is/are your income sources? [MULTIPLE ANSWERS] | Salary | 1 |
|  |  | Selling/business | 2 |
|  |  | Husband’s support | 3 |
|  |  | Relative’s support | 4 |
|  |  | Other (Specify___) | 96 |
| 2.8 | What is your average monthly household income in the past 12 months in KHR? | Record as mentioned | N/A |
| 2.9 | How long have you stayed in this village? [IN MONTHS] | Record as mentioned | N/A |

**3. Household durable assets**

| **N** | **Items** | **Quantity (if no=0; don’t know=97)** |
| --- | --- | --- |
|  | Radio | Number: |
|  | Television | Number: |
|  | Non-mobile telephone | Number: |
|  | Mobile telephone (non-smartphone) | Number: |
|  | Smart Phone | Number: |
|  | Computer | Number: |
|  | Refrigerator | Number: |
|  | Table | Number: |
|  | Chair | Number: |
|  | Bed | Number: |
|  | Sewing machine | Number: |
|  | Clock | Number: |
|  | Sofa | Number: |
|  | Wardrobe | Number: |
|  | Generator, battery, or solar panel | Number: |
|  | Video/VCD/DVD player/recorder | Number: |
|  | Bicycle | Number: |
|  | Animal-drawn cart | Number: |
|  | Motorcycle or motor scooter | Number: |
|  | Car or truck | Number: |
|  | Boat with a motor | Number: |
|  | Motorcycle cart | Number: |
|  | Boat without a motor | Number: |
|  | Water pump | Number: |
|  | Other (Specify___) | Number: |

**4. Access to media**

| **N** | **Question** | **Answer** | **Code** |
| --- | --- | --- | --- |
| 4.1 | How often did you read newspapers or magazines in the past week? | Not at all | 0 |
|  |  | Less than once a week | 1 |
|  |  | At least once a week | 2 |
| 4.2 | How often did you listen to the radio in the past week? | Not at all | 0 |
|  |  | Less than once a week | 1 |
|  |  | At least once a week | 2 |
| 4.3 | How often did you watch television in the past week? | Not at all | 0 |
|  |  | Less than once a week | 1 |
|  |  | At least once a week | 2 |
| 4.4 | How often did you have access to the internet in the past week? | Not at all | 0 |
|  |  | Less than once a week | 1 |
|  |  | At least once a week | 2 |
| 4.5 | Do you own a smartphone?  [THE PHONE THAT CAN GET AN INTERNET CONNECTION.] | Not at all | 0 |
|  |  | Less than once a week | 1 |
|  |  | At least once a week | 2 |
| 4.6 | Do you usually use any social media (e.g., Facebook, Tik Tok, Instagram, Twitter, Telegram, etc.)? | Not at all | 0 |
|  |  | Less than once a week | 1 |
|  |  | At least once a week | 2 |
| 4.7 | For what purpose do you use social media platforms? [MULTIPLE ANSWERS] | Entertainment | 1 |
|  |  | News | 2 |
|  |  | Health news | 3 |
|  |  | Contact with friends and relatives | 4 |
|  |  | Work | 5 |
|  |  | Other (Specify ___) | 96 |

**5. Maternal Outcomes, Antenatal Care (ANC)**

| **N** | **Question** | **Answer** | **Code** |
| --- | --- | --- | --- |
| 5.1 | Did you have any ANC visits at the health facility during your last pregnancy? | No | 0 (Skip to Q 5.6) |
|  |  | Yes | 1 |
| 5.2 | If yes, when did the 1^st^ ANC take place?  [RECORD ACCORDING TO WEEK OF PREGNANCY] | Record as mentioned | N/A |
| 5.3 | If yes, how many ANC visits did you complete in total? | Record as mentioned: _____ | N/A |
| 5.4 | If yes, from whom did you receive your LAST ANC? | VHSG | 1 |
|  |  | TBA | 2 |
|  |  | A skilled provider | 3 |
|  |  | Could not remember | 97 |
|  |  | Others | 96 |

| **5.5 If yes, did you have your ANC visit at a health facility in ___ of delivery (No=0, Yes=1, 2)?**  **[ASK FOR PINK BOOK FROM PARTICIPANT. IF THE PARTICIPANT DOES NOT HAVE THE BOOK, SKIP THIS QUESTION.]** | **1^st^** | **2^nd^** | **3^rd^** | **4^th^** | **…** |
| --- | --- | --- | --- | --- | --- |
| Less than 12 weeks |  |  |  |  |  |
| 20-24 weeks |  |  |  |  |  |
| 30-32 weeks |  |  |  |  |  |
| 36-38 weeks |  |  |  |  |  |
| … |  |  |  |  |  |

| **N** | **Question** | **Answer** | **Code** |
| --- | --- | --- | --- |
| 5.6 | In your last pregnancy, were you given | | |
|  | 5.6a. Tetanus toxoid | (No / Yes) | 0 / 1 |
|  | 5.6b. 90 iron-folic acid tablets | (No / Yes) | 0 / 1 |
|  | 5.6c. One dose of deworming medicine | (No / Yes) | 0 / 1 |
|  | 5.6d. HIV test | (No / Yes) | 0 / 1 |
|  | 5.6e. Syphilis test | (No / Yes) | 0 / 1 |
| 5.7 | If yes to 5.6a and 5.6b, **how many doses** did you receive during your last pregnancy? |  |  |
|  | 5.7a. Tetanus toxoid | Record as mentioned | N/A |
|  | 5.7b. 90 iron-folic acid tablets | Record as mentioned | N/A |
| 5.8 | If yes to 5.6a and 5.6b, **when** were you given during your last pregnancy? [RECORD ACCORDING TO WEEK OF PREGNANCY] |  |  |
|  | 5.8a. Tetanus toxoid | Record as mentioned | N/A |
|  | 5.8b. 90 iron-folic acid tablets | Record as mentioned | N/A |
| 5.9 | Were there any complications during your last pregnancy?  [CHECK WITH MOTHER WHETHER SHE WAS TOLD BY THE HEALTH PROVIDER ABOUT COMPLICATIONS.] | No (Skip to Q 5.11) | 0 |
|  |  | Yes | 1 |
| 5.10 | If so, from whom did you seek care for the most recent complication? | VHSG | 1 |
|  |  | A skilled provider | 2 |
|  |  | Nurse | 3 |
|  |  | TBA | 4 |
|  |  | Did not remember | 97 |
|  |  | Other (Specify___) | 96 |
| 5.11 | In your last pregnancy, how many times were you visited at home OR called by someone other your your family/friends? [IF NONE, RECORD 0] | Record as mentioned | N/A |
| 5.12 | Who visited you? | VHSG | 1 |
|  |  | A skilled provider | 2 |
|  |  | Nurse | 3 |
|  |  | TBA | 4 |
|  |  | Other (Specify___) | 96 |
| 5.13 | When was the last visit by VHSG during your pregnancy? | Record week of pregnancy | N/A |

**6. Maternal Outcomes, Delivery**

| **N** | **Question** | **Answer** | **Code** |
| --- | --- | --- | --- |
| 6.1 | When was your last delivery? (Month and Year)  Don’t know month=6666  Don’t know year=9999 | Record as mentioned | N/A |
| 6.2 | At what week of your pregnancy did you deliver?  [MEASURE IN WEEK: E.G., 38] | Record as mentioned | N/A |
| 6.3 | Where did your last delivery take place? | Home | 1 |
|  |  | Public health facilities | 2 |
|  |  | Private health facilities | 3 |
|  |  | Other (Specify___) | 96 |
| 6.4 | Who delivered your last baby? | No one | 0 |
|  |  | A skilled midwife | 1 |
|  |  | A traditional birth attendant | 2 |
|  |  | A nurse | 3 |
|  |  | A doctor | 4 |
|  |  | Other (Specify___) | 96 |
| 6.5 | After your last delivery, how many days did you stay at the health facility? [IF DELIVERED AT HOME, PLEASE WRITE HOME] | Record as mentioned | N/A |
| 6.6 | What was the weight of your last baby right after birth?  [RECORD IN KILOGRAM] | Record as mentioned | N/A |
| 6.7 | Did you experience complicated delivery? | No (Skip to Q 7.1) | 0 |
|  |  | Yes | 1 |
| 6.8 | If yes, what were the complications? [MORE THAN ONE ANSWER] | Record as mentioned | N/A |

**7. Maternal Outcomes, Postnatal Care (PNC)**

| **N** | **Question** | **Answer** | | **Code** |
| --- | --- | --- | --- | --- |
| 7.1 | Did you have any PNC visit after delivery in your last pregnancy? | No | | 0 |
|  |  | Yes (Skip to Q 7.3) | | 1 |
| 7.2 | If yes, skip this question.  If no, what were the reasons that you did not seek PNC? [MULTIPLE ANSWERS] | Not affordable | | 1 |
|  |  | Not accessible | | 2 |
|  |  | Too busy (work, children) | | 3 |
|  |  | Self-treated | | 4 |
|  |  | Facility has a poor infrastructure | | 5 |
|  |  | Facility poorly stocked | | 6 |
|  |  | Poor staff attitude | | 7 |
|  |  | Poor staff knowledge | | 8 |
|  |  | Poor quality of acre | | 9 |
|  |  | Service not available | | 10 |
|  |  | No transportation | | 11 |
|  |  | Did not need | | 12 |
|  |  | Inconvenient hours | | 13 |
|  |  | Long waiting times | | 14 |
|  |  | Prefer home care | | 15 |
|  |  | Family didn’t want me to go | | 16 |
|  |  | Unaware of PNC | | 17 |
|  |  | Other (Specify______) | | 96 |
| 7.3 | If yes, when did your first PNC take place after the delivery? [RECORD IN DAYS, WEEKS, OR MONTHS AFTER DELIVERY] | Record as mentioned | | N/A |
| 7.4 | If yes, how many PNC visits did you complete so far? | Record as mentioned | | N/A |
| 7.5 | Did you receive counseling about: ____ during any PNC visits? | | | |
|  | 7.5a Body hygiene | | (No / Yes) | 0 / 1 |
|  | 7.5b Hand washing | | (No / Yes) | 0 / 1 |
|  | 7.5c Nutrition for mother | | (No / Yes) | 0 / 1 |
|  | 7.5d Breastfeeding | | (No / Yes) | 0 / 1 |
|  | 7.5e Risk symptoms for both mother and newborn | | (No / Yes) | 0 / 1 |
| 7.6 | If yes, from whom did you receive the counseling? | | Record as mentioned | N/A |
|  |  |  | Yes | 1 |
| 7.7 | Were there any **complications** within the first month of your last delivery? [NEED TO GIVE SOME EXAMPLES HERE] | | No (Skip to Q 8.1) | 0 |
|  |  |  | Yes | 1 |
| 7.8 | If so, from whom did you seek care?  [MULTIPLE ANSWERS] | | VHSG | 1 |
|  |  |  | A skilled provider | 2 |
|  |  |  | Nurse | 3 |
|  |  |  | TBA | 4 |
|  |  |  | Did not remember | 97 |
|  |  |  | Other (Specify___) | 96 |

**8. *Neonatal (0-28 days) Outcomes***

*The following questions refer to when your LAST child was between 0 and 28 days old.*

| **N** | **Question** | **Answer** | **Code** |
| --- | --- | --- | --- |
| 8.1 | Have you ever breastfed your last child? | No (Skip to Q 8.3) | 0 |
|  |  | Yes | 1 |
| 8.2 | If yes, how long after birth did you first put your last child to the breast? [IF LESS THAN 1 HOUR, RECORD ‘0' HOURS. IF LESS THAN 24 HOURS, RECORD HOURS. OTHERWISE, RECORD DAYS.] | Record as mentioned | N/A |
| 8.3 | If not, what was the reason? | Record as mentioned | N/A |
| 8.4 | Did your child receive a Hepatitis B vaccination after delivery? | No (Skip to Q 8.6) | 0 |
|  |  | Yes | 1 |
| 8.5 | If yes, when did **your child** receive the vaccine after birth? [IDEALLY WITHIN 24 HOURS] [RECORD IN HOURS] | Record as mentioned | N/A |
| 8.6 | Did **your child** receive BCG vaccination after delivery? | No (Skip to Q 8.8) | 0 |
|  |  | Yes | 1 |
| 8.7 | If yes, when did your child receive the BCG vaccination? [IDEALLY WITHIN 24 HOURS] [RECORD IN HOURS] | Record as mentioned | N/A |
| 8.8 | Was your child **examined** with risk symptoms (e.g., convulsion, fast or slow breathing, etc.) | No (Skip to Q 8.10) | 0 |
|  |  | Yes | 1 |
|  |  | Do not know | 97 |
| 8.9 | If yes, was your child been referred to see the doctor? | No | 0 |
|  |  | Yes | 1 |
|  |  | Do not know | 97 |
| 8.10 | Did a VHSG visit your child after returning home post-delivery? | No (Skip to Q 8.16) | 0 |
|  |  | Yes | 1 |
| 8.11 | If yes, when did that take place? [RECORD WITHIN HOURS, DAYS, OR WEEKS OF DELIVERY] | Record as mentioned | N/A |
| 8.12 | Did VHSG examine your baby during the visit? | No | 0 |
|  |  | Yes | 1 |
| 8.13 | If yes, how satisfied were you with the examination? | Very satisfied | 1 |
|  |  | Satisfied | 2 |
|  |  | Neutral | 3 |
|  |  | Dissatisfied | 4 |
|  |  | Very dissatisfied | 5 |
| 8.14 | Did you receive counseling about caring for your baby from VHSG during the home visit? | No (Skip to Q8.16) | 0 |
|  |  | Yes | 1 |
| 8.15 | If yes, how satisfied were you with the counseling? | Very satisfied | 1 |
|  |  | Satisfied | 2 |
|  |  | Neutral | 3 |
|  |  | Dissatisfied | 4 |
|  |  | Very dissatisfied | 5 |
| 8.16 | Did your child have complications within the first month of birth? | No (Skip to Q9.1) | 0 |
|  |  | Yes | 1 |
| 8.17 | If yes, from whom did you seek care?  [IF DID NOT SEEK CARE, RECORD “NO”] | Record as mentioned | N/A |

**9. *Young Infant (2-12 months) Outcomes***

*The following questions refer to when your LAST child was between 2 and 12 months old.*

| **N** | **Question** | **Answer** | **Code** |
| --- | --- | --- | --- |
| 9.1 | How many months did you breastfeed your last child? [RECORD IN MONTHS] | Record as mentioned | N/A |
| 9.2 | During the first 6 weeks after delivery, did your child receive ___ vaccination?  [CHECK/ASK FOR THE CHILD’S VACCINATION CARD—THE YELLOW CARD] | | |
|  | 9.2a. Dyphteria Tetanus Pertussis – HepB-Hib (DTP-HepB-Hib 1) | (No / Yes) | 0 / 1 |
|  | 9.2b. Oral Polio Vaccine #1 (OPV1) | (No / Yes) | 0 / 1 |
|  | 9.2c. Pneumococcal Conjugate Vaccine#1 (PCV1) | (No / Yes) | 0 / 1 |
| 9.3 | During the first 2.5 months of birth, did your child receive ____vaccination?  [CHECK THE CHILD’S VACCINATION CARD] | | |
|  | 9.3a. Dyphteria Tetanus Pertussis – HepB-Hib (DTP-HepB-Hib 2) | (No / Yes) | 0 / 1 |
|  | 9.3b. Oral Polio Vaccine #2 (OPV2) | (No / Yes) | 0 / 1 |
|  | 9.3c. Pneumococcal Conjugate Vaccine#2 (PCV2) | (No / Yes) | 0 / 1 |
| 9.4 | During the first 3.5 months of birth, did your child receive ____vaccination? [CHECK THE CHILD’S VACCINATION CARD] | | |
|  | 9.4a. Dyphteria Tetanus Pertussis – HepB-Hib (DTP-HepB-Hib 3) | (No / Yes) | 0 / 1 |
|  | 9.4b. Oral Polio Vaccine #3 (OPV3) | (No / Yes) | 0 / 1 |
|  | 9.4c. Pneumococcal Conjugate Vaccine#3 (PCV3) | (No / Yes) | 0 / 1 |
|  | 9.4d. Inactivated polio vaccine (IPV) | (No / Yes) | 0 / 1 |
| 9.5 | During the first 6 months after birth, did your child receive ___ vaccination?  [CHECK THE CHILD’S VACCINATION CARD] | | |
|  | 9.5a. Vitamin A 100,000 UI | (No / Yes) | 0 / 1 |
|  | 9.5b. Japanese encephalitis (JE) | (No / Yes) | 0 / 1 |
| 9.6 | During the first 12 months after birth, did your child receive ___ vaccination?  [CHECK THE CHILD’S VACCINATION CARD] | | |
|  | 9.6a. Vitamin A 200,000 UI | (No / Yes) | 0 / 1 |
|  | 9.6b. One dose of deworming | (No / Yes) | 0 / 1 |
| 9.7 | Did your child have diarrhea within the last two weeks?  [PAST TWO WEEKS OF THIS INTERVIEW] | No (Skip to Q9.12) | 0 |
|  |  | Yes | 1 |
| 9.8 | From whom did you receive ORS? | Did not receive | 0 |
|  |  | VHSG | 1 |
|  |  | Nurse | 2 |
|  |  | Doctor | 3 |
|  |  | Other (Specify) | 96 |
| 9.9 | From whom did you seek any care for your child? | Did not seek care | 0 |
|  |  | VHSG | 1 |
|  |  | Nurse | 2 |
|  |  | Doctor | 3 |
|  |  | Other (Specify) | 96 |
| 9.10 | Was your child given Zinc supplements? | No | 0 |
|  |  | Yes | 1 |
| 9.11 | If yes, who gave your child Zinc supplements? | VHSG | 1 |
|  |  | Nurse | 2 |
|  |  | Doctor | 3 |
|  |  | Other (Specify) | 96 |
| 9.12 | Did your child have ARI/fever within the last two weeks?  [PAST TWO WEEKS OF THIS INTERVIEW] | No | 0 |
|  |  | Yes | 1 |
| 9.13 | Has your child received a growth monitoring check and follow-up? | No | 0 |
|  |  | Yes | 1 |
|  |  | Do not know | 97 |
| 9.14 | Was there any harm/side effect suffered due to treatment or advice provided by health staff? | No | 0 |
|  |  | Yes | 1 |
| 9.15 | Was there any harm/side effect suffered due to treatment or advice provided by VHSG? | No | 0 |
|  |  | Yes | 1 |

**10. *Young Child (13-24 months) Outcomes***

*The following questions refer to when your LAST child was between 13 and 24 months old.*

| **N** | **Question** | **Answer** | **Code** |
| --- | --- | --- | --- |
| 10.1 | How many months did you breastfeed your last child? [IF THE WOMAN IS CURRENTLY BREASTFEEDING, ASK HOW LONG SHE HAS BEEN DOING UP TO THE TIME OF THE INTERVIEW. RECORD IN MONTHS] | Record as mentioned | N/A |
| 10.2 | Were you reminded about birth spacing? | No | 0 |
|  |  | Yes | 1 |
| 10.3 | If yes, who reminded you about birth spacing? | Family/relative | 1 |
|  |  | VHSG | 2 |
|  |  | Nurse | 3 |
|  |  | Doctor | 4 |
|  |  | Other (Specify__) | 96 |
| 10.4 | Has your child received a growth monitoring check and follow-up? | No | 0 |
|  |  | Yes | 1 |
|  |  | Do not know | 97 |
| 10.5 | During the first 13 and 24 months after birth, did your child receive ___ vaccination? [CHECK/ASK FOR THE CHILD’S VACCINATION CARD—THE YELLOW CARD] | | |
|  | 10.5a. Vitamin A 100,000 UI (twice) | (No / Yes) | 0 / 1 |
|  | 10.5b. One dose of deworming (twice) | (No / Yes) | 0 / 1 |
| 10.6 | Between 13 and 24 months after birth, were your child/did your child | | |
|  | 10.6a. Examined with the risk symptoms (e.g., convulsion, fast or slow breathing, etc.) | (No / Yes) | 0 / 1 |
|  | 10.6b. Receive a growth monitoring check and follow-up | (No / Yes) | 0 / 1 |
|  | 10.6c. Experience medium or severe malnutrition | (No / Yes) | 0 / 1 |
|  | 10.6d. Receive an HIV quick test | (No / Yes) | 0 / 1 |

***11.***  ***Health Insurance Coverage, Social Health Protection, and Health Expenditure,***

| **N** | **Question** | **Answer** | **Code** |
| --- | --- | --- | --- |
| 11.1 | Did any health insurance cover you? | No | 0 |
|  |  | Yes | 1 |
| 11.2 | Did any health insurance cover your newborn or infant? | No | 0 |
|  |  | Yes | 1 |
| 11.3 | What kind of health insurance did you have for yourself and/or your newborn or infant? | Record as mentioned | N/A |
| 11.4 | Has this household been identified as poor through the Identification of Poor Households (IDPoor)? | No (Skip to Q 11.6) | 0 |
|  |  | Yes | 1 |
| 11.5 | If yes, ask to see the equity, priority access card and other cards, including post-identification | Equity Card - Yes card seen | 1 |
|  |  | Equity Card - Yes card not seen | 2 |
|  |  | Priority access card - Yes card seen | 3 |
|  |  | Priority access card - Yes card not seen | 4 |
| 11.6 | Do members of this household receive free or subsidized health care that other people would normally have to pay for? | No (Skip to Q 11.8) | 0 |
|  |  | Yes, Free | 1 |
|  |  | Yes, Subsidized | 2 |
|  |  | Other (Specify____) | 96 |
| 11.7 | What free or subsidized health care has any member of this household received? | Health equity funds | 1 |
|  |  | Health Insurance through Employer (e.g., National Social Security Fund) | 2 |
|  |  | Other Privately Purchased Commercial Health Insurance | 3 |
|  |  | Other (Specify___) | 96 |
| 11.8 | How much in total was spent on transport to and return from the health facility where you received treatment for the pregnancy-related complications? [IF DID NOT EXPERIENCE COMPLICATION, RECORD “0”] | Record as mentioned (in KHR) | N/A |
| 11.9 | How much did you spend on seeking advice and treatment at the health facility where you received treatment for pregnancy-related complications? [IF DID NOT EXPERIENCE COMPLICATION, RECORD “0”] | Record as mentioned (in KHR) | N/A |
| 11.10 | Were there any other expenses incurred during your trip to the health facility where you received treatment for pregnancy-related complications? | No (Skip to Q 11.13) | 0 |
|  |  | Yes | 1 |
| 11.11 | If yes, how much was incurred? | Record as mentioned (in KHR) | N/A |
| 11.12 | Where did the money come from to pay for the cost of visiting the health facility where you received treatment for pregnancy-related complications? [MULTIPLE ANSWER] | Did not experience any complications | 0 |
|  |  | Out of pocket | 1 |
|  |  | Health equity funds | 2 |
|  |  | Voucher | 3 |
|  |  | Fee exemption | 4 |
|  |  | NGOs support | 5 |
|  |  | National social security fund | 6 |
|  |  | Employer-based insurance | 7 |
|  |  | Privately purchased commercial health insurance | 8 |
|  |  | Other (Specify___) | 96 |
| 11.13 | How much in total did you spend to receive ANC?  [IF DID NOT HAVE ANC VISIT, RECORD “0”, THEN SKIP TO Q11.15] | Record as mentioned (in KHR) | N/A |
|  | a. How much was spent on transport? | Record as mentioned (in KHR) | N/A |
|  | b. How much was the tests/check-ups? | Record as mentioned (in KHR) | N/A |
| 11.14 | Where did the money come from to receive ANC?  [MULTIPLE ANSWER] | Did not receive ANC | 0 |
|  |  | Out of pocket | 1 |
|  |  | Health equity funds | 2 |
|  |  | Fee exemption | 3 |
|  |  | NGOs support | 4 |
|  |  | National social security fund | 5 |
|  |  | Employer-based insurance | 6 |
|  |  | Privately purchased commercial health insurance | 7 |
|  |  | Other (Specify___) | 96 |
| 11.15 | How much in total did you spend to deliver the baby? | Record as mentioned (in KHR) | N/A |
|  | a. How much was the transport? | Record as mentioned (in KHR) | N/A |
|  | b. How much was the delivery? | Record as mentioned (in KHR) | N/A |
| 11.16 | Where did the money come from to deliver the baby?  [MULTIPLE ANSWER] | Out of pocket | 1 |
|  |  | Health equity funds | 2 |
|  |  | Fee exemption | 3 |
|  |  | NGOs support | 4 |
|  |  | National social security fund | 5 |
|  |  | Employer-based insurance | 6 |
|  |  | Privately purchased commercial health insurance | 7 |
|  |  | Other (Specify___) | 96 |
| 11.17 | How much in total did you spend to receive PNC?  [IF DID NOT HAVE PNC VISIT, RECORD “0”, THEN SKIP TO Q11.19] | Record as mentioned (in KHR) | N/A |
|  | a. How much was spent on the transport? | Record as mentioned (in KHR) | N/A |
|  | b. How much was spent on the tests? | Record as mentioned (in KHR) | N/A |
| 11.18 | Where did the money come from to receive PNC? | Did not receive PNC | 0 |
|  |  | Out of pocket | 1 |
|  |  | Health equity funds | 2 |
|  |  | Fee exemption | 3 |
|  |  | NGOs support | 4 |
|  |  | National social security fund | 5 |
|  |  | Employer-based insurance | 6 |
|  |  | Privately purchased commercial health insurance | 7 |
|  |  | Other (Specify___) | 96 |
| 11.19 | How much was spent on transport to and return from the health facility where your newborn or infant received check-ups and treatment? | Record as mentioned (in KHR) | N/A |
| 11.20 | How much in total was spent on seeking advice and treatment at the health facility, where your newborn or infant received check-up and treatment? | Record as mentioned (in KHR) | N/A |
| 11.21 | How much did you spend on other expenses during your trip to the health facility where your newborn or infant received check-ups and treatment? | Record as mentioned (in KHR) | N/A |
| 11.22 | Where did the money come from to pay for the cost of visiting the health facility where your newborn or infant received treatment? | Did not receive any treatment | 0 |
|  |  | Out of pocket | 1 |
|  |  | Health equity funds | 2 |
|  |  | Fee exemption | 3 |
|  |  | NGOs support | 4 |
|  |  | National social security fund | 5 |
|  |  | Employer-based insurance | 6 |
|  |  | Privately purchased commercial health insurance | 7 |
|  |  | Other (Specify___) | 96 |
| 11.23 | In your household, who has the final say on health expenditure? | Myself | 1 |
|  |  | My husband/partner | 2 |
|  |  | My relatives | 3 |
|  |  | My parents | 4 |
|  |  | Other (specify) | 96 |

**Anthropometry Measure (Baseline/Midline/Endline)**

Household ID:**__ __ __ __**

Date of the interview: **__/__/____** Interviewer ID: **__ __** Participant ID: **__ __ __ __**

| **Anthropometry measures from birth to 2 years** | | | |
| --- | --- | --- | --- |
|  | **Child 1** | **Child 2** | **Child 3** |
| [Record same child order (youngest = 01, second youngest = 02, etc.)] | \|___\|___\| | \|___\|___\| | \|___\|___\| |
| Q Age | \|___\|___\| Months | \|___\|___\| Months | \|___\|___\| Months |
| Q Sex | Female/Male | Female/Male | Female/Male |
| Q [First time:  Record weight in kilograms (Kg)] | Kg \|___\|___\| **.** \|___\|___\| | Kg \|___\|___\| **.** \|___\|___\| | Kg \|___\|___\| **.** \|___\|___\| |
| Q4. [Second time:  Record weight in kilograms (Kg)] | Kg \|___\|___\| **.** \|___\|___\| | Kg \|___\|___\| **.** \|___\|___\| | Kg \|___\|___\| **.** \|___\|___\| |
| *[Continue with height measurement below]* | | | |
| Q5. [First time:  Record height in centimeters (Cm)] | Cm \|___\|___\|___\| **.** \|___\| | Cm \|___\|___\|___\| **.** \|___\| | Cm \|___\|___\|___\| **.** \|___\| |
| Q6. [Second time:  Record height in centimeters (Cm)] | Cm \|___\|___\|___\| **.** \|___\| | Cm \|___\|___\|___\| **.** \|___\| | Cm \|___\|___\|___\| **.** \|___\| |
| Q7. [Third time: Only if difference between measurements >1.0cm | Cm \|___\|___\|___\| **.** \|___\| | Cm \|___\|___\|___\| **.** \|___\| | Cm \|___\|___\|___\| **.** \|___\| |
| Q8. Confirm the child laying or standing for measurements | 1. Laying 2. Standing | 1. Laying 2. Standing | 1. Laying 2. Standing |
| Q9. Check if (child’s name) has edema | 1. Yes 2. No | 1. Yes 2. No | 1. Yes 2. No |

This is the end of the questionnaire.

Thank you very much for participating in our study.
