# Supplementary material for: i-MoMCARE: Innovative Mobile Technology for Maternal and Child Health Care in Cambodia—study protocol of a cluster randomized controlled trial
Source: Trials. 2023 Oct 26;24:692. doi: 10.1186/s13063-023-07724-z (PMC10601211; doi:10.1186/s13063-023-07724-z)
Supplement: Supplementary file 3 — Additional file 3. Qualitative tools. [file 13063_2023_7724_MOESM3_ESM.docx]

**TOPIC GUIDE FOR IN-DEPTH INTERVIEW**

(With health center staff for gap analysis)

**Project title:** *i-MoMCARE* – Innovative Mobile Technology for Maternal and Child Health Care in Cambodia: study protocol of a cluster randomized controlled trial

**To be completed by the interviewer:**

Interview date: ______/ _______/ ____________

Interviewer’s initials: _________________________

Participant’s ID: _____________________________________

Time interview started: ________________ Time interview ended: _________________

| **Domain** | **Questions** | **Prompts** |
| --- | --- | --- |
| Roles and Responsibilities | 1. Please tell us about your roles in your current position. |  |
|  | 1. How long have you been in this position?​ (months/years) |  |
|  | 1. What did you do before this job? |  |
|  | 1. Could you walk us through your daily job? |  |
|  | 1. What motivates you to do your work? |  |
| Supervision and support for health center staff | 1. What kind of support does the health center provide to staff (like yourself)? | - Are there any guidelines to follow? - What types of training, especially MCH-related? - How often is the training? - % of participation of the health center staff in training? - Type of equipment/resources available for health center staff? |
|  | 1. How does the health center supervise your work? | - Are there any guidelines to follow? - Number of working hours per day and days per week - Are there any challenges? - How do you address those challenges? |
|  | 1. How does the health center assess the performance of health center staff? | - How do they perform? Provide examples of performance indicators/measurements - % of referred cases, e.g., complications, danger signs, child illness - Are there any policies/ guidelines to follow? |
|  | 1. Does the health center provide you with cash/in-kind support? | - How do you get paid? Performance-based? Monthly? - Were there any delays? - How satisfied are you with the support? - Are there any policies/ guidelines to follow? |
| Supervision and support for village health support groups (VHSGs) | 1. What kind of support does the health center provide to VHSGs? | - Are there any guidelines to follow? - What type of training, especially MCH-related? - How often is the training? - % of participation of the VHSGs in training? - Type of equipment/resources available for VHSGs? |
|  | 1. How does the health center supervise VHSGs’ work? | - Are there any guidelines to follow? - Number of working hours per day and days per week - Are there any challenges? - How do you address those challenges? |
|  | 12. How does the health center assess the performance of VHSGs? | - How do they perform? Provide examples of performance indicators/measurements - % of home visits by VHSGs - % of referred cases (e.g., complications, danger signs, child illnesses) - Are there any policies/guidelines to follow? |
|  | 13. Does the health center provide VHSGs with cash/in-kind support? | - How do VHSGs get paid? Performance-based? Monthly? - Were there any delays? - How satisfied are VHSGs with the support? - Are there any policies/ guidelines to follow? |
| Digital Health and Literacy | 14. How involved are you with the technology/information communication technology (ICT)? | What about other health center staff? |
|  | 15. What is your overall digital or mobile literacy? | What about other health center staff? |
|  | 16. Was there any ICT-related training provided to health center staff? | What is your level of willingness to learn? And among other health center staff? |
|  | 17. Was there any ICT-related training provided to VHSGs? | What is the level of willingness to learn among VHSGs? |
|  | 18. Have you heard of mobile or digital health (using mobile phones or other wireless technology in medical care)? | If the answer is no, please give an example of i-MoMCARE to them |
|  | 19. What do you think of digital health to improve MCH via VHSGs’ work? |  |
|  | 20. What do you think of the app and its application to your work? | What could be the potential challenges of using digital health in your work? |
|  | 21. How acceptable is using the app/phone among VHSGs as part of their work? |  |
| Policy, challenges, and recommendation | 22. Are you aware of any ICT integration and data sharing in health care in Cambodia? | If there is, how would the policy support or hinder projects like i-MoMCARE? |
|  | 23. What is/are the major challenges you face at work? | - Availability of staff - Staff competency - Quality of care provided - Interpersonal relations, etc. |
|  | 24. Are there any recommendations you would like to give to make your work more efficient? |  |

This is the end of the interview.

Thank you very much for your participation.

**TOPIC GUIDE FOR IN-DEPTH INTERVIEW**

(With village health support group for gap analysis)

**Project title:** *i-MoMCARE* – Innovative Mobile Technology for Maternal and Child Health Care in Cambodia: study protocol of a cluster randomized controlled trial

**To be completed by the interviewer:**

Interview date: ______/ _______/ ____________

Interviewer’s initials: _________________________

Participant’s ID: _____________________________________

Time interview started: ________________ Time interview ended: _________________

| **Domain** | **Question** | **Prompts** |
| --- | --- | --- |
| Demographics | 1. Age: Please indicate your age by choosing one of the following age ranges (20-29, 30-39, 40-49, 50-59, or 60-69) |  |
|  | 1. Marital status: e.g., married, never married, divorced, widowed |  |
|  | 1. Numbers of children: e.g., Do you have any children? How many? | Do you have grandchildren? |
|  | 1. How would you define your ethnicity: |  |
|  | 1. Education: how many years did you go to school? | Do you have a degree/title? |
|  | 1. Work: Have you had a job before becoming a VHSG? | If yes, what was it? |
| Roles or services | 1. How long have you been a village health support group (VHSG)? |  |
|  | 1. How did you become a VHSG? | - Were you recruited? - Volunteered? |
|  | 1. Have you received any training to become a VHSG? | - If yes, tell me more about it - Have you received any follow-up training? (continuous training) |
|  | 10. Can you describe your role as a VHSG? What do you typically do? Can you walk me through your job from start to finish? | - Recruitment of pregnant women and children under 24 months - Follow-ups - Traveling to meet beneficiaries - Referrals - ANC, delivery, PNC - Medication distribution - Vaccination - Checking and/or recording danger signs - Non-compliant patients, etc. |
|  | 11. Do you like your role as a VHSG? | - What are the pros and cons? - What motivates you to do your work? - What de-motivates/prevents you from doing your job? |
|  | 12. What do you think about the workload? | - What/who determines your workload? - Are you able to complete all tasks? - Have you received any incentives? From whom? How are the incentives calculated? - Is it fair in your opinion? - Private family matters (emergency in own family, husband/mother-in-law doesn’t want her to work, household work that needs to be done, etc.)? Different objectives? Disagreements with co-workers? |
| Training, support, and constraints | 13. Could you describe any types of MCH-related training that you received in the last 12 months? | - Who were those trainers? - Were they health center staff? - Were the training planned and regularly conducted? - Were they sufficient for you to perform your tasks (e.g., determine danger signs of illnesses or complications)? - How much were you able to understand the content of the training? - Were the contents difficult to understand? - Were you satisfied with the training provided? |
|  | 14. Could you describe the types of support/resources you receive from the health center you work for? | Were they regular/on time and sufficient? |
|  | 15. Could you elaborate on obstacles, if any, when implementing your roles as VHSG to assist pregnant women and children in need of health services? | Personal and external barriers or challenges? |
| Supervision and support | 16. How do the health center staff supervise your work? | - Are there any guidelines? - Number of working hours per day and days per week |
|  | 17. How do health center staff assess your performance? | - How well did you perform? - Please provide examples of performance, indicators/ measurements, % of home visits by you, % of referred cases (e.g., complications, danger signs, child illnesses) - Are there any policies/guidelines to follow? |
|  | 18. Does health center provide you with cash/in-kind support? | - How do you get paid? - Performance-based? Monthly? - Any delay? - How satisfied are you with the support? |
| Collaboration and data collection | 19. Are there other VHSGs in your area village? | - - If yes, how many?   - Do you work together? |
|  | 1. Do you work with health center staff? | - Tell me how you work together - Scheduling home visits? - Task allocation? |
|  | 21. How do you currently collect data on patients? | - - Paperwork? Pros? Cons?   - Are there any guidelines to follow?   - Mode of communication?   - Number of working hours per day and days per week |
|  | 22. How are you reporting information back to the health center? | - - How often do you need to report back to the health center?   - How do you find the process? (easy, tedious, etc.) |
| Technology | 23. Do you own a phone?  (Is it a smartphone?) | - - If not, does anyone in your household own a mobile phone, which you can use?   - If not, have you ever used a phone before, occasionally?   - If yes, are you on social media (e.g., Facebook…)? How much time do you spend on your smartphone daily? Do you find it easy to use? |
|  | 24. Would a mobile phone be useful for counseling patients and collecting data instead of pen and paper? | Describe how it works.  Potential pros/cons? |
|  | 25. Would you be willing to learn? | If no, why not? |
| Challenges and recommendations Closing remarks | 26. Do you wish you could change anything about your job? | If yes, please share.  (knowledge, structure, workload, compensation) |
|  | 27. Are there pregnant women and children in your area who a VHSG does not visit? | - - If yes, why do you think that is?   - What can be done to change this? |
|  | 28. What are the challenges you face at work? | How do you address those challenges? |
|  | 29. Do you have any recommendations to improve your current work? | - - To make your job easier/more efficient?   - To improve MCH services in your area? |

This is the end of the interview.

Thank you very much for your participation.

**TOPIC GUIDE FOR IN-DEPTH INTERVIEW**

(With health center staff for the pilot, midline, and endline)

**Project title:** *i-MoMCARE* – Innovative Mobile Technology for Maternal and Child Health Care in Cambodia: study protocol of a cluster randomized controlled trial

**To be completed by the interviewer:**

Interview date: ______/ _______/ ____________

Interviewer’s initials: _________________________

Participant’s ID: _____________________________________

Time interview started: ________________ Time interview ended: _________________

| **Domain** | **Questions** | **Prompts** |
| --- | --- | --- |
| Training, user experience, and satisfaction | 1. How long have you used the desktop application (the web) of i-MoMCARE? |  |
|  | 2. How did you feel about the training provided before using the web in your work? | Was the training sufficient? If not, why not? |
|  | 3. Please tell me your overall experience using the web. | - - How easy or difficult is it to use it?   - Please tell us more about the difficulties that you experienced using the web. |
|  | 4. How acceptable was using the web to perform your task? | Would you continue using this technology in your work if it is available? |
|  | 5. In your opinion, what do you think of the usefulness of the web to your job? | - Please provide examples. - Could you better track VHSGs’ progress and detect complicated cases early? |
|  | 6. What is your overall satisfaction with the web? | - If not satisfied, what could be done differently? - Please tell us your experience of the level of satisfaction of the web. |
| Barriers to usage and recommendation | 7. Were there any features you wish were available on the web? |  |
|  | 8. What do you think of the technical support provided to you before joining the program? | - - Was it sufficient?   - Was there anything you wish could be done differently? |
|  | 9. What do you think of the features of the web? | - - How relevant were they?   - How useful do you think they are in aiding your job?   - Is there anything you wish could be done differently? |
|  | 10. Was there any feedback from other VHSGs or health center staff on the web who did not use the app? | If yes, please share. |
|  | 11. Is there anything that would be helpful for us to consider for improving the design/content of the web? | If yes, please share (feature and content). |
|  | 12. Would you recommend we incorporate mobile app services in MCH work in other places in Cambodia? | What about other health programs? |
|  | 13. What did you like about being a part of this program? What did you not like about it? |  |
|  | 14. What were some of the challenges you faced during the program? | What are some of the issues with the web ( e.g., internet reception, your digital literacy)? |
|  | 15. Is there any feedback you would like to give to improve the overall program (i-MoMCARE)? |  |

This is the end of the interview.

Thank you very much for your participation.

**TOPIC GUIDE FOR IN-DEPTH INTERVIEW**

(With village health volunteer groups for the pilot, midline, and endline)

**Project title:** *i-MoMCARE* – Innovative Mobile Technology for Maternal and Child Health Care in Cambodia: study protocol of a cluster randomized controlled trial

**To be completed by the interviewer:**

Interview date: ______/ _______/ ____________

Interviewer’s initials: _________________________

Participant’s ID: _____________________________________

Time interview started: ________________ Time interview ended: _________________

| **Domain** | **Questions** | **Prompts** |
| --- | --- | --- |
| Demographics | 1. Age: Please indicate your age by choosing one of the following age ranges: 20-29, 30-39, 40-49, 50-59, or 60-69 |  |
|  | 2. Marital status: e.g., are you married, never married, divorced, widowed |  |
|  | 3. Numbers of children: e.g., Do you have any children? How many? | Do you have grandchildren? |
|  | 4. How would you define your ethnicity? |  |
|  | 5. Education: How many years did you go to school? | Do you have a degree/title? |
|  | 1. Work: Have you had a job before becoming a VHSG? | If yes, what was it? |
| Training, user experience, and satisfaction | 7. How long have you used the mobile application of i-MoMCARE? | How did you feel about the training provided before using the app in your work? |
|  | 8. Please tell me your overall experience using the app. | - - How easy or difficult is it to use it?   - Please tell us more about the difficulties that you experienced using the app.   - How acceptable was it to you to use the app to perform your task?   - Would you continue using this technology in your work if it is available? |
|  | 9. What do you think of the usefulness of the app to your job? | - - Please provide examples   - Could you better manage your home visit schedule and refer cases to the health center by using the app? |
|  | 10. What is your overall satisfaction with the app? | - - If not satisfied, what could be done differently?   - Please tell us your experience with the level of satisfaction of the app. |
| Barriers to technology usage and recommendations | 1. Were there any features that you wish were available in the app? |  |
|  | 12. What do you think of the technical support provided to you before joining the program? | - - Was it sufficient?   - Was there anything you wish could be done differently? |
|  | 13. What do you think of multimedia or behavior-change communication content? | - How relevant were they? - How useful do you think they are in aiding your job? - Is there anything you wish could be done differently? |
|  | 14. Were there any feedback you received from the beneficiaries regarding the use of the app? | If yes, please share. |
|  | 15. Were there any feedback from other VHSGs who did not use the app on it? | If yes, please share. |
|  | 16. Is there anything that would be helpful for us to consider in terms of better design/content the app? | If yes, please share.  (feature and content) |
|  | 17. Would you recommend we incorporate mobile app services in MCH work in other places in Cambodia? | What about other health programs? |
|  | 18. What did you like about being a part of this program? What did you not like about it? |  |
|  | 19. What were some of the challenges you faced during the program? | What are some of the issues with the app itself (mobile phone, internet reception, your digital literacy)? |
|  | 20. Is there any feedback you would like to give to improve the overall program (i-MoMCARE)? |  |

This is the end of the interview.

Thank you very much for your participation.

**TOPIC GUIDE FOR IN-DEPTH INTERVIEW**

(With health center directors for the pilot, midline, and endline)

**Project title:** *i-MoMCARE* – Innovative Mobile Technology for Maternal and Child Health Care in Cambodia: study protocol of a cluster randomized controlled trial

**To be completed by the interviewer:**

Interview date: ______/ _______/ ____________

Interviewer’s initials: _________________________

Participant’s ID: _____________________________________

Time interview started: ________________ Time interview ended: _________________

| **Domain** | **Question** | **Prompts** |
| --- | --- | --- |
| Role and Responsibility | - 1. How long have you been the health center director? | What did you do prior to this role? |
|  | 2. What are your major roles/responsibilities as the health center director? |  |
|  | 3. How many staff are working in your health center? |  |
| Feasibility and acceptability of the mobile and web application | 5. Through your observation of the i-MoMCARE implementation, how do you find the practical application of mobile and web applications at your workplace? | What are your opinions on the acceptability, usefulness, and helpfulness of the apps to your work? |
|  | 6. Has the mobile or web application addressed any challenges (e.g., work arrangement, schedule, etc.) that your health center has faced? | Would you appreciate the continued presence of the program if funder continues the program? |
|  | 7. Have you observed any improvement in the number of referred cases related to MCH and attributed to the presence of the i-MoMCARE project? | Could you provide some examples that you have observed? |
|  | 8. What challenges have arisen from using this technology in your work? | What about your staff and VHSGs? |
|  | 9. Have you observed or heard clients'/beneficiaries’ and health center staff’s feedback about i-MoMCARE? | Could you tell us about those accounts you heard? (both positive and negative) |
| Village health volunteer (VHSG)and health center (HC) staff’s workload, efficiency, and effectiveness | 10. How has the workload for VHSG/HC staff in i-MoMCARE changed over the program? | Has it increased? Decreased? |
|  | 11. What is your opinion on VHSG/HC staff efficiency during i-MoMCARE implementation? |  |
|  | 12. What is your opinion on VHSG/HC staff effectiveness during the program? |  |
| Digital literacy, reflection, and recommendation | 13. What is your opinion on your staff’s digital literacy before i-MoMCARE? |  |
|  | 14. What about after i-MoMCARE? | Has it improved? |
|  | 15. How did i-MoMCARE impact your work? The health center? What about your staff? Including VHSGs? | Pros and cons |
|  | 16. Have you received any feedback from the beneficiaries regarding i-MoMCARE? | If yes, please share. |
|  | 17. Were there any feedback from other VHSGs or health center staff who did not use the app on them? | If yes, please share. |
|  | 18. Is there anything that would be helpful for us to consider for future digital health projects like this one? | If yes, please share.  (feature and content) |
|  | 19. Would you recommend we incorporate mobile app services in MCH work in other places in Cambodia? | What about other health programs? |
|  | 20. What did you like about being a part of this program? What did you not like about it? |  |
|  | 21. Is there any feedback you would like to give to improve the overall program? |  |

This is the end of the interview.

Thank you very much for your participation.

**TOPIC GUIDE FOR KEY INFORMANT INTERVIEW**

(With health center directors for gap analysis)

**Project title:** *i-MoMCARE* – Innovative Mobile Technology for Maternal and Child Health Care in Cambodia: study protocol of a cluster randomized controlled trial

**To be completed by the interviewer:**

Interview date: ______/ _______/ ____________

Interviewer’s initials: _________________________

Participant’s ID: _____________________________________

Time interview started: ________________ Time interview ended: _________________

| **Domain** | **Questions** | **Prompts** |
| --- | --- | --- |
| Roles and responsibilities | 1. Could you tell us about your roles in your current position? |  |
|  | 2. How long have you been in this position?​ (months/years) |  |
|  | 3. What did you do prior to this job? |  |
|  | 4. Could you walk us through your daily job? |  |
|  | 5. What motivates you to do your work? |  |
| Service provision | 6. Could you tell us about the types of health services that your health center offers? | - - How many villages are there in your HC catchment area?   - Total population (male/female)?   - What are the total population of pregnant women and <24 months children? |
|  | 7. How extensive is the coverage of maternal and child health services? | - - The proportion of those who completed four or more antenatal care visits   - # of identification of danger sign illness/complication   - Number of delivery at health center by a skilled midwife   - Number of post-partum counseling (PNC)   - Number of identification of sick newborns   - Number of neonates receiving tetanus toxoid |
|  | 8. Are there any village health volunteer groups (VHVGs) in your health center? | - - If so, how many?   - How closely do you work with VHSGs? |
|  | 9. What do you think of the role of VHSGs? | How vital are VHSGs in providing health services to the target groups successfully? |
| Supervision and support for health center (HC) staff | 10. What kind of support does the health center provide to health center staff? | - - Are there any guidelines to follow?   - What kind of training, especially MCH-related?   - How often is the training?   - % of participation of the health center staff in training?   - Type of equipment/resources available for health center staff? |
|  | 11. How does the health center supervise health center staff’s work? | - Are there any guidelines to follow? - Number of working hours per day and days per week - Are there any challenges? - How do you address those challenges? |
|  | 12. How does the health center assess the performance of health center staff? | - - How do they perform? Provide examples of performance indicators/measurements   - % of referred cases, e.g., complications, danger signs, child illness   - Are there any policies/ guidelines to follow? |
|  | 13. Does the health center provide health center staff cash/in-kind support? | - - How do VHSG get paid? Performance-based? Monthly?   - Were there any delays?   - How satisfied are health center staff with the support?   - Are there any policies/guidelines to follow? |
| Supervision and support for village health volunteer groups (VHSGs) | 14. What kind of support does the health center provide to VHSGs? | - - Are there any guidelines to follow?   - What kind of training, especially MCH-related?   - How often is the training?   - % of participation of the VHSGs in training?   - Type of equipment/resources available for VHSGs? |
|  | 15. How does the health center supervise VHSGs’ work? | - - Are there any guidelines to follow?   - Number of working hours per day and days per week   - Are there any challenges?   - How do you address those challenges? |
|  | 16. How does the health center assess the performance of VHSGs? | - - How do they perform? Provide examples of performance indicators/measurements   - % of home visits by VHSGs   - % of referred cases, e.g., complications, danger signs, child illnesses   - Are there any policies/ guidelines to follow? |
|  | 17. Does the health center provide VHSGs with cash/in-kind support? | - How do VHSGs get paid? Performance-based? Monthly? - Were there any delays? - How satisfied are VHSGs with the support? - Are there any policies/ guidelines to follow? |
| Digital health literacy | 18. How involved are your staff with the technology/ information communication technology (ICT)? | What about VHSGs? |
|  | 19. What is the overall digital or mobile literacy of your staff? | How many of them are using a smartphone or a computer? |
|  | 20. Was there any ICT-related training provided to HC staff? | What is the level of willingness to learn among your HC staff? |
|  | 21. Was there any ICT-related training provided to VHSGs? | What is the level of willingness to learn among VHSGs? |
|  | 22. Have you heard of mobile health or digital health (using mobile phones or other wireless technology in medical care)? | If the answer is no, please give an example of i-MoMCARE to them. |
|  | 23. What do you think of digital health to improve MCH via VHSGs’ work? |  |
|  | 24. What do you think of the app and its application to your work? | What could be the potential challenges of using digital health in your work? |
|  | 25. How acceptable is using the app/phone among VHSGs as part of their work? |  |
| Policy, challenges, and recommendation | 26. Are you aware of any ICT integration and data sharing in health care in Cambodia? | If there is, how would the policy support or hinder projects like i-MoMCARE? |
|  | 27. What is/are the major challenges you face at work? | Staff availability, staff competency, quality of care, interpersonal relations, etc. |
|  | 28. Are there any recommendations you would like to give to make your work more efficient? |  |

This is the end of the interview.

Thank you very much for your participation.

**TOPIC GUIDE FOR KEY INFORMANT INTERVIEW**

(With NMCHC and NGOs for gap analysis)

**Project title:** Innovative Mobile Technology for Maternal and Child Health Care in Cambodia (*i-MoMCARE*): A Cluster Randomized Controlled Trial

**To be completed by the interviewer:**

Interview date: ______/ _______/ ____________

Interviewer’s initials: _________________________

Participant’s ID: _____________________________________

Time interview started: ________________ Time interview ended: _________________

| **Domain** | **Question** | **Prompts** |
| --- | --- | --- |
| Roles and responsibilities | 1. Please introduce yourself briefly | Please tell us about your current position. |
|  | 2. How long have you been in this position? |  |
|  | 3. What did you do before this role? |  |
|  | 4. What does your daily work look like? | What are your roles and responsibilities? |
| Service provision and funding | 5. What health services does your institution provide? |  |
|  | 6. How long has your institution been providing the services? |  |
|  | 7. Who are the beneficiaries? | Geography and the total number of beneficiaries (could it be approximative?) |
|  | 8. What is the coverage population? |  |
|  | 9. And where are they located? |  |
|  | 10. How extensive is the coverage for maternal and child health (MCH) services? | - - Coverage (%) of 4+ ANC visits to HCs in rural areas   - # of identification of danger sign illness/complication   - # of delivery at HC by primary or secondary midwives   - # of post-partum counseling (PNC)   - # of identification of sick newborns   - # of neonates receiving tetanus toxoid |
|  | 11. How involved are you with the health service providers, such as the health center? |  |
|  | 12. What are the primary funding sources for your work? |  |
|  | 13. How closely do you/your institution work with other health service providers, NGOs etc.? |  |
| Overall policy context | 14. Are there policies/guidelines/plans to increase MNCH coverage in the coming years? | - Focus on the rural areas - Key actors, especially frontline implementers - Are there any plan to incorporate health technology, such as mHealth, into the current policy/guideline in the future? |
|  | 15. Are there any programs to support pregnant women and mothers of children under five to get access to the nearest health center? |  |
|  | 16. Are you aware of any ICT integration and data sharing in health care in Cambodia? | If there is, how would the policy support or hinder i-MoMCARE? |
| Digital health literacy | 17. How involved are your staff with information communication technology? |  |
|  | 18. What is the overall digital or mobile literacy of your staff? | What is the percentage of those who use a smartphone or a computer? |
|  | 19. Was there any information communication technology-related training provided to staff in the last 12 months? | - - If yes, what is the training?   - What was the level of willingness to learn among your staff? |
|  | 20. Have you heard of digital health (using mobile phones or other wireless technology in medical care)? | If the answer is no, please give an example of i-MoMCARE to them. |
|  | 21. What is your opinion on digital health? |  |
|  | 22. What do you think of using digital health to improve MCH work in the community? | - Will the app be useful for your work? - What could be the potential challenges of using the phone app in your work? |
|  | 23. How acceptable are you of using the apps to improve your work? |  |
| Challenges and recommendations | 24. What are the major challenges you face at work? | - Funding - Availability of staff - Staff competency - Quality of care provided - Interpersonal relations, etc. |
|  | 25. Are there any recommendations you would like to give to make your work more efficient? |  |

This is the end of the interview.

Thank you very much for your participation.

**TOPIC GUIDE FOR KEY INFORMANT INTERVIEW**

(With provincial health departments and operational districts for gap analysis)

**Project title:** *i-MoMCARE* – Innovative Mobile Technology for Maternal and Child Health Care in Cambodia: study protocol of a cluster randomized controlled trial

**To be completed by the interviewer:**

Interview date: ______/ _______/ ____________

Interviewer’s initials: _________________________

Participant’s ID: _____________________________________

Time interview started: ________________ Time interview ended: _________________

| **Domain** | **Questions** | **Prompts** |
| --- | --- | --- |
| Roles and responsibilities | 1. Could you tell us about your roles in your current position? |  |
|  | 2. How long have you been in this position?​ (months/years) |  |
|  | 3. What did you do prior to this job? |  |
|  | 4. Could you walk us through your daily job? |  |
|  | 5. What motivates you to do your work? |  |
| Service provision | 6. Could you tell us about the types of health services that your institution offers? | - How many HC are there in your catchment area? - Total population (male/female)? - What are the total population of pregnant women and <24 months children? |
|  | 7. How extensive is the coverage for maternal and child health (MCH) services? |  |
| Supervision and support for health centers | 8. How does your institution assess the performance of health center? | Are there any guidelines to follow? |
|  | 9. What kind of support does your institution provide to the health centers? |  |
|  | 10. How does the reporting system work between your institution and the health center? | - Are there any challenges? - How do you address those challenges? |
| Digital health literacy | 11. How involved are your staff with the technology/information communication technology (ICT)? |  |
|  | 12. What is the overall digital or mobile literacy of your staff? | How many of them are using a smartphone or a computer? |
|  | 13. Was there any ICT-related training provided to your staff? | What is the level of willingness to learn among your staff? |
|  | 14. Have you heard of mobile or digital health (using mobile phones or other wireless technology in medical care)? | If the answer is no, please give an example of i-MoMCARE to them |
|  | 15. What do you think of digital health to improve MCH via VHSGs’ work? |  |
|  | 16. What do you think of the app and its application to your work? | What could be the potential challenges of using digital health in your work? |
|  | 17. Are there any digital health projects your institution is currently a part of? | If yes, please elaborate (project type, duration, funding, beneficiaries) |
|  | 18. Are there any other digital health projects your institution will participate in the near future? | If yes, please elaborate? Project type, duration, funding, beneficiaries, etc. |
| Policy, challenges, and recommendation | 19. Are you aware of any ICT integration and data sharing in health care in Cambodia? | If there is, how would the policy support or hinder projects like i-MoMCARE? |
|  | 20. What are the significant challenges you face at work? | - Availability of staff - Staff competency - Quality of care provided - Interpersonal relations, etc. |
|  | 21. Are there any recommendations you would like to give to make your work more efficient? |  |

This is the end of the interview.

Thank you very much for your participation.

**TOPIC GUIDE FOR FOCUS GROUP DISCUSSION**

(With pregnant women for after pilot, midline, and endline)

**Project title:** *i-MoMCARE* – Innovative Mobile Technology for Maternal and Child Health Care in Cambodia: study protocol of a cluster randomized controlled trial

**To be completed by the interviewer:**

Interview date: ______/ _______/ ____________

Interviewer’s initials: _________________________

Time interview started: ________________ Time interview ended: _________________

| Participant ID | Age | Employment | Education | Marital status | Total number of children | Number of under-five children |
| --- | --- | --- | --- | --- | --- | --- |
| 1 |  |  |  |  |  |  |
| 2 |  |  |  |  |  |  |
| … |  |  |  |  |  |  |

| **Domain** | **Question** | **Prompts** |
| --- | --- | --- |
| Maternal and child health (MCH) services | 1. Could you tell us about your experience seeking MCH services during and after your last pregnancy? | - Motivation to - Barriers to - Support for seeking MCH services |
|  | 2. Why did you choose the health center for MCH services? | Besides the health center, did you go elsewhere for MCH services? |
|  | 3. What do you think of the overall MCH services you received from the health center? | Satisfactory? |
|  | 4. What is your opinion on VHSG support during (ANC) and after (PNC) your current/last pregnancy? | - Were they helpful? - What would you say about your relationship with the VHSG who assisted your ANC) and PNC? |
|  | 5. Did you notice that VHSGs used a mobile application to schedule, follow up, and show short video clips to you? | What makes you notice that? Did the VHSGs tell you? |
|  | 6. What do you think of the short video clips that VHSGs showed you during the visit? |  |
|  | 7. How effective are those videos compared to a series of photos? |  |
|  | 8. In your opinion, what did VHSGs do well? | What about things that could be improved? |
|  | 9. Were there any complications during your pregnancy? | From whom did you seek help? |
|  | 10. Could you tell us about your experience receiving support from VHSGs who used and did not use the mobile application? | Did you find the mobile app helpful for yourself (e.g., schedule on time, visit the health center as needed, etc.)? |
| Challenges and recommendations | 11. What were your top three challenges during your pregnancy and delivery? | How did you address those challenges? |
|  | 12. Could you tell us about the perception regarding the feasibility and acceptability of the mobile app that VHSGs used? | - Would you prefer using support from VHSGs using the mobile app than that from VHSGs without the mobile app? - Did you notice any differences between the two VHSG groups? - Would you recommend other mothers in your village and other villages using support from VHSGs who use the mobile app? |
|  | 13. Is there any recommendation you would like to give to improve the program (i-MoMCARE)? |  |

This is the end of the interview.

Thank you very much for your participation.

**TOPIC GUIDE FOR FOCUS GROUP DISCUSSION**

(With pregnant women for gap analysis)

**Project title:** Innovative Mobile Technology for Maternal and Child Health Care in Cambodia (*i-MoMCARE*): A Cluster Randomized Controlled Trial

**To be completed by the interviewer:**

Interview date: ______/ _______/ ____________

Interviewer’s initials: _________________________

Time interview started: ________________ Time interview ended: _________________

| Participant ID | Age | Employment | Education | Marital status | Total number of children | Number of under two children |
| --- | --- | --- | --- | --- | --- | --- |
| 1 |  |  |  |  |  |  |
| 2 |  |  |  |  |  |  |
| … |  |  |  |  |  |  |

| **Domain** | **Questions** | **Prompts** |
| --- | --- | --- |
| Maternal and child health (MCH) services | 1. Could you tell us about your experience of seeking MCH services during pregnancy and after delivery? | - Motivation to - Barriers to - Support for seeking MCH services |
|  | 2. Where did you seek MCH services during your last pregnancy? | Public and private health facilities |
|  | 3. Why did or didn’t you choose to come to the health center for MCH services? | Besides the health center, did you go elsewhere for MCH services? |
|  | 4. What do you think of the overall MCH services you received? | From the health center or other health facilities? |
|  | 5. Who assisted you the most during your last pregnancy, excluding friends and family? | Nurses, health center staff, VHSG, doctors, etc. |
|  | 6. What is your opinion on those who assisted you during your last pregnancy? | - - Were they helpful?   - What would you say about your relationship with the one who assisted you during your last pregnancy? |
|  | 7. In your opinion, what did they do well? | What about things that could be improved? |
|  | 8. Were there any complications during your pregnancy? | From whom did you seek help? |
|  | 9. How many ANC visits did you do during your last pregnancy? | - - Do you know the minimum recommended ANC and PNC visits?   - If the total visit did not meet the minimum recommended (4ANC and 10 PNC), ask why not? |
|  | 10. How many PNC visits did you do after your last pregnancy? |  |
|  | 10. Who usually accompanied you to those visits? | Is it your husband/partner, friends, or relatives? |
| Challenges and recommendations | 11. What were your top three challenges during your pregnancy/delivery? | How did you address those challenges? |
|  | 12. What support did you wish to get during and after your pregnancy?  From whom did you wish to receive the support? | From whom did you wish to receive the support? |
|  | 13. Is there any recommendation you would like to give to help your next pregnancy/other pregnant women? |  |

This is the end of the interview.

Thank you very much for your participation.
