# Supplementary material for: i-MoMCARE: Innovative Mobile Technology for Maternal and Child Health Care in Cambodia—study protocol of a cluster randomized controlled trial
Source: Trials. 2023 Oct 26;24:692. doi: 10.1186/s13063-023-07724-z (PMC10601211; doi:10.1186/s13063-023-07724-z)
Supplement: Supplementary file 4 — Additional file 4. PIS & informed consent forms. [file 13063_2023_7724_MOESM4_ESM.docx]

**Participant Information Sheet & Consent Forms**

(For pregnant women and mothers)

**Protocol title:** *i-MoMCARE* – Innovative Mobile Technology for Maternal and Child Health Care in Cambodia: A Cluster Randomized Controlled Trial

**Principal Investigator and co-investigators, with the contact number and organization:**

- Principal Investigator: Dr. Yi Siyan (MD, MHSc, PhD), Saw Swee Hock School of Public Health, National University of Singapore, Email: [siyan@nus.edu.sg](mailto:siyan@nus.edu.sg) /Tel: +65 8753 3823
- Co-Principal Investigator: Prof. Saphonn Vonthanak (MD, MSc, PhD), University of Health Sciences, Cambodia Email: [vonthanak@uhs.edu.kh](mailto:vonthanak@uhs.edu.kh)/Tel: (+855) 92 222 679
- Co-Investigator: Dr. Kim Ratana (MD, MPH), National Maternal and Child Health Center, Cambodia Email: [rattanamch@gmail.com](mailto:rattanamch@gmail.com)/Tel: (+855) 12 880 745
- Co-Investigator: Assist. Prof. Mam Sovatha (BSc, BBA, MPH), University of Health Sciences, Cambodia, Email: [sovatha@uhs.edu.kh](mailto:sovatha@uhs.edu.kh)/Tel: (+855) 16 869 695

**What is the purpose of this research?**

This randomized controlled trial aims to deploy and evaluate the feasibility and acceptability of innovative mobile technology for maternal and child health care in Cambodia. The mobile health technology will provide job aid to the village health support groups to more effectively schedule, determine illness and complications, and refer mothers and children to health centers for prenatal, delivery, and postnatal care.

**Who can participate in the research? What is the expected duration of my participation? What is the duration of this research?**

You are eligible to participate in the study if you (1) are a woman of reproductive age (aged between 18 and 49), (2) are a woman who is currently pregnant or has the last child under two years of age, (3) are a woman who lived in the village in the past 6 months, and (4) received pregnancy-related services from the health center and support from village health support groups during pregnancy. The data collection will take approximately one hour for a focus group discussion and 30 to 45 minutes for an interview. This is a 3-year research project.

**What is the approximate number of research participants involved?**

This study needs approximately 2,000 participants.

**What will be done if I take part in this research study?**

If you agree to participate in this study, we will conduct a focus group discussion or interview with you and several other mothers and pregnant women in your community. We will ask about your health conditions and experience seeking health care from village health support groups and health centers during pregnancy, delivery, and after delivery. You can answer and discuss these questions with other peers in your peers during the focus group discussions. The research team will transcribe the recordings, remove identifying information, and discard the recordings and questionnaires after the study.

With your permission, the interviewer or discussion moderator will audio-record and take notes during the discussion and interview. The purpose of recording is to accurately record the information you provide. If you choose not to be recorded, I will take notes instead. If you agree to be recorded but feel uncomfortable during the discussion, I can turn off the recorder at your request. You can stop the discussion or interview anytime if you do not wish to continue. Discussion and interview quotations and recordings might be used anonymously in reports.

**How will my privacy and the confidentiality of my research records be protected?**

Neither your name nor identifiable information will be collected or linked to your responses. All information and audio files will be stored on a central computer to protect your identity and the confidentiality of your information. Access to this information will be strictly controlled with a security code system. By signing the Informed Consent Form attached, you (or your legally acceptable representative, if relevant) are authorizing (1) collection, access to, use, and storage of your personal data and (2) disclosure to authorized service providers and relevant third parties.

“Personal Data” means data about you that makes you identifiable from such data or other information an organization has or is likely to have access to. This includes medical conditions, medications, investigations, and treatment history. Information and “Personal Data” collected for this study will be confidential. To the extent of the applicable laws and regulations, your records will not be publicly available.

Data collected and entered in the case report forms are the property of the research team. In the event of any publication regarding this study, your identity will remain confidential.

By participating in this study, you confirm that you have read, understood, and consent to the Personal Data Protection Notification available at (<http://www.nus.edu.sg/opc/personal-data-protection/nus-data-protection-policy>).

**What are the possible discomforts and risks for participants?**

Participating in this study has no perceivable risks as only interviews will be conducted. However, potential discomforts could be related to psychological distress or uneasiness during the discussions with your peers or researchers. You can refuse to answer any questions you feel uncomfortable with and quit the study at any time without giving any reasons if you feel uncomfortable.

**What is the compensation for any injury?**

There is no compensation for any injury as this study involves interviews only. By signing this consent form, you will not waive your legal right or release the parties involved in this study from the liability for negligence.

**Will there be reimbursement for participation?**

A token of appreciation (USD 2.50) will be reimbursed for your time and effort in this discussion.

**What are the possible benefits to me and others?**

There is no direct benefit to you for participating in this research study. However, certain benefits may be derived from any medical research study. Such benefits include the information learned during this study may help improve maternal and child health services for you and your community.

**Can I refuse to participate in this research?**

Your participation in this study is entirely voluntary. You may refuse to participate or discontinue participation at any time without penalty or loss of benefits to which you would normally be entitled. Your decision to participate in the study will not affect your relationship with your community, authorities, or health facilities.

**Whom should I call if I have any questions or problems?**

If you have any questions about the research, please do not hesitate to contact Mrs. Suy Sovanthida, Project Coordinator, Tel: (+855) 12 986 814/E-mail: [sovanthidasuy@uhs.edu.kh](mailto:sovanthidasuy@uhs.edu.kh) or Dr. Ung Meng Ieng, Research Fellow, Tel: (+855) 12 838 172/E-mail: [mung@nus.edu.sg](mailto:mung@nus.edu.sg).

**Consent Form**

**Protocol title:** *i-MoMCARE* – Innovative Mobile Technology for Maternal and Child Health Care in Cambodia: A Cluster Randomized Controlled Trial

**Principal Investigator with the contact number and organization:**

Dr. Yi Siyan (MD, MHSc, PhD), Assistant Professor, Saw Swee Hock School of Public Health, National University of Singapore, Email: [siyan@nus.edu.sg](mailto:siyan@nus.edu.sg)/Tel: +65 8753 3823

I hereby acknowledge that:

1. I have agreed to take part in the above research.
2. I have received a copy of this information sheet explaining my data use in this research. I understand its contents and agree to donate my data for the use of this research.
3. I can withdraw from the research by informing the Principal Investigator or interviewer, and all my data will be discarded.
4. I will not have any financial benefits from this research's commercial development.
5. I agree to the audio recording of my participation in the research.
6. I agree for my quotes to be used in any publication or presentation relating to this research, which will be attributed with a pseudonym/anonymously.

**_______________________________ ___________**

Name and Signature (Participant) Date

**_______________________________ ___________**

Name and Signature (Consent Taker) Date

**Participant Information Sheet & Consent Forms**

(For healthcare providers)

**Protocol title:** *i-MoMCARE* – Innovative Mobile Technology for Maternal and Child Health Care in Cambodia: A Cluster Randomized Controlled Trial

**Principal Investigator and co-investigators, with the contact number and organization:**

- Principal Investigator: Dr. Yi Siyan (MD, MHSc, PhD), Saw Swee Hock School of Public Health, National University of Singapore, Email: [siyan@nus.edu.sg](mailto:siyan@nus.edu.sg) /Tel: +65 8753 3823
- Co-Principal Investigator: Prof. Saphonn Vonthanak (MD, MSc, PhD), University of Health Sciences, Cambodia Email: [vonthanak@uhs.edu.kh](mailto:vonthanak@uhs.edu.kh)/Tel: (+855) 92 222 679
- Co-Investigator: Dr. Kim Ratana (MD, MPH), National Maternal and Child Health Center, Cambodia Email: [rattanamch@gmail.com](mailto:rattanamch@gmail.com)/Tel: (+855) 12 880 745
- Co-Investigator: Assist. Prof. Mam Sovatha (BSc, BBA, MPH), University of Health Sciences, Cambodia, Email: [sovatha@uhs.edu.kh](mailto:sovatha@uhs.edu.kh)/Tel: (+855) 16 869 695

**What is the purpose of this research?**

This randomized controlled trial aims to deploy and evaluate the feasibility and acceptability of innovative mobile technology for maternal and child health care in Cambodia. The mobile health technology will provide job aid to the village health support groups to more effectively schedule, determine illness and complications, and refer mothers and children to health centers for prenatal, delivery, and postnatal care.

**Who can participate in the research? What is the expected duration of my participation? What is the duration of this research?**

You are eligible to participate in the study if you are (1) a staff member at the National Maternal and Child Health Center, Provincial Health Department, Operational District, health center, or village health support group; (2) aged 18 and older; and (3) have been in the position for at least one year. The data collection will take approximately one hour for a focus group discussion and 30 to 45 minutes for an interview. This is a 3-year research project.

**What is the approximate number of research participants involved?**

This study needs approximately 2,000 participants.

**What will be done if I take part in this research study?**

If you agree to participate in this study, we will conduct a focus group discussion or interview with you and several other mothers and pregnant women in your community. We will ask about your health conditions and experience seeking health care from village health support groups and health centers during pregnancy, delivery, and after delivery. You can answer and discuss these questions with other peers in your peers during the focus group discussions. The research team will transcribe the recordings, remove identifying information, and discard the recordings and questionnaires after the study.

With your permission, the interviewer or discussion moderator will audio-record and take notes during the discussion and interview. The purpose of recording is to accurately record the information you provide. If you choose not to be recorded, I will take notes instead. If you agree to be recorded but feel uncomfortable during the discussion, I can turn off the recorder at your request. You can stop the discussion or interview anytime if you do not wish to continue. Discussion and interview quotations and recordings might be used anonymously in reports.

**How will my privacy and the confidentiality of my research records be protected?**

Neither your name nor identifiable information will be collected or linked to your responses. All information and audio files will be stored on a central computer to protect your identity and the confidentiality of your information. Access to this information will be strictly controlled with a security code system. By signing the Informed Consent Form attached, you (or your legally acceptable representative, if relevant) are authorizing (1) collection, access to, use, and storage of your personal data and (2) disclosure to authorized service providers and relevant third parties.

“Personal Data” means data about you that makes you identifiable from such data or other information an organization has or is likely to have access to. This includes medical conditions, medications, investigations, and treatment history. Information and “Personal Data” collected for this study will be confidential. To the extent of the applicable laws and regulations, your records will not be publicly available.

Data collected and entered in the case report forms are the property of the research team. In the event of any publication regarding this study, your identity will remain confidential.

By participating in this study, you confirm that you have read, understood, and consent to the Personal Data Protection Notification available at (<http://www.nus.edu.sg/opc/personal-data-protection/nus-data-protection-policy>).

**What are the possible discomforts and risks for participants?**

Participating in this study has no perceivable risks as only interviews will be conducted. However, potential discomforts could be related to psychological distress or uneasiness during the discussions with your peers or researchers. You can refuse to answer any questions you feel uncomfortable with and quit the study at any time without giving any reasons if you feel uncomfortable.

**What is the compensation for any injury?**

There is no compensation for any injury as this study involves interviews only. By signing this consent form, you will not waive your legal right or release the parties involved in this study from the liability for negligence.

**Will there be reimbursement for participation?**

A token of appreciation (USD 2.50) will be reimbursed for your time and effort in this discussion.

**What are the possible benefits to me and others?**

There is no direct benefit to you for participating in this research study. However, certain benefits may be derived from any medical research study. Such benefits include the information learned during this study may help improve maternal and child health services for you and your community.

**Can I refuse to participate in this research?**

Your participation in this study is entirely voluntary. You may refuse to participate or discontinue participation at any time without penalty or loss of benefits to which you would normally be entitled. Your decision to participate in the study will not affect your relationship with your community, authorities, or health facilities.

**Whom should I call if I have any questions or problems?**

If you have any questions about the research, please do not hesitate to contact Mrs. Suy Sovanthida, Project Coordinator, Tel: (+855) 12 986 814/E-mail: [sovanthidasuy@uhs.edu.kh](mailto:sovanthidasuy@uhs.edu.kh) or Dr. Ung Meng Ieng, Research Fellow, Tel: (+855) 12 838 172/E-mail: [mung@nus.edu.sg](mailto:mung@nus.edu.sg).

**Consent Form**

**Protocol title:** *i-MoMCARE* – Innovative Mobile Technology for Maternal and Child Health Care in Cambodia: A Cluster Randomized Controlled Trial

**Principal Investigator with the contact number and organization:**

Dr. Yi Siyan (MD, MHSc, PhD), Assistant Professor, Saw Swee Hock School of Public Health, National University of Singapore, Email: [siyan@nus.edu.sg](mailto:siyan@nus.edu.sg)/Tel: +65 8753 3823

I hereby acknowledge that:

1. I have agreed to take part in the above research.
2. I have received a copy of this information sheet explaining my data use in this research. I understand its contents and agree to donate my data for the use of this research.
3. I can withdraw from the research by informing the Principal Investigator or interviewer, and all my data will be discarded.
4. I will not have any financial benefits from this research's commercial development.
5. I agree to the audio recording of my participation in the research.
6. I agree for my quotes to be used in any publication or presentation relating to this research, which will be attributed with a pseudonym/anonymously.

**_______________________________ ___________**

Name and Signature (Participant) Date

**_______________________________ ___________**

Name and Signature (Consent Taker) Date
